# Five-Year Absolute Risk–Based and Age-Based Breast Cancer Screening in the US

**DOI:** 10.1001/jamanetworkopen.2025.52944

**Published:** 2026-01-20

**Authors:** Oguzhan Alagoz, Yifan Lu, Eugenio Gil Quessep, Karla Kerlikowske, Jeanne S. Mandelblatt, Brian L. Sprague, Amy Trentham-Dietz, John Hampton, Rick Groeneweg, Harry J. de Koning, Diana L. Miglioretti, Clyde B. Schechter, Nicolien T. van Ravesteyn, Anna N. A. Tosteson, Natasha K. Stout, Kathryn P. Lowry

**Affiliations:** 1 Department of Industrial and Systems Engineering, University of Wisconsin–Madison; 2 Department of Public Health, Erasmus MC, University Medical Center Rotterdam, the Netherlands; 3 Department of Medicine, University of California at San Francisco; 4 Department of Epidemiology & Biostatistics, University of California at San Francisco; 5 Department of Oncology, Georgetown University Medical Center, Georgetown University, Washington, DC; 6 Department of Surgery and the University of Vermont Cancer Center, Larner College of Medicine, University of Vermont, Burlington; 7 Department of Population Health Sciences and the Carbone Cancer Center, School of Medicine and Public Health, University of Wisconsin–Madison; 8 Department of Public Health Sciences, University of California at Davis; 9 Kaiser Permanente Washington Health Research Institute, Seattle; 10 Department of Family and Social Medicine, Albert Einstein College of Medicine, Bronx, New York; 11 Geisel School of Medicine, Dartmouth College, and Dartmouth Cancer Center, Lebanon, New Hampshire; 12 Division of Cancer Control and Population Sciences, National Cancer Institute, Bethesda, Maryland; 13 Department of Radiology, University of Washington School of Medicine, Fred Hutchinson Cancer Center, Seattle

## Abstract

**Importance:**

General mammography screening guidelines target women at average risk within a specified age range (age based) and do not consider absolute risk of individual women at a given age (risk based).

**Objective:**

To compare outcomes of mammography screening strategies that vary by 5-year risk of invasive breast cancer vs age-based strategies.

**Design, Setting, and Participants:**

This decision analytical model used 2 established Cancer Intervention and Surveillance Modeling Network (CISNET) breast cancer models and simulated US women born in 1980 who were aged 40 years or older without a prior history of breast cancer. Modeling analyses were conducted from April 2023 to April 2025.

**Intervention:**

Digital breast tomosynthesis delivered via 50 screening strategies (3 age based and 47 risk based) vs a no-screening scenario. Five-year absolute invasive breast cancer risk was based on the validated Breast Cancer Surveillance Consortium, version 3 calculator. Women’s 5-year breast cancer risk was categorized as low, average, intermediate, or high.

**Main Outcomes and Measures:**

Primary outcomes included lifetime number of breast cancer deaths averted and false-positive screening recalls. Lifetime outcomes were averaged across models and expressed per 1000 women screened.

**Results:**

Nine risk-based screening strategies were associated with a comparable or greater number of deaths averted than biennial age-based screening from ages 40 to 74 years (B40-74) (range across strategies for mean model estimates, 6.8-7.5 per 1000 women vs 6.8 per 1000 women) as well as reduced false-positive recalls by 8% to 23% (1050-1257 per 1000 women for risk-based screening strategies vs 1365 per 1000 women for B40-74). For example, a risk-based approach using a combination of biennial screening (for women at low risk aged 55-74 years, at average risk aged 50-59 years, at intermediate risk aged 45-54 years, and at high risk aged 40-49 years) and annual screening (for women at average risk aged 60-74 years, at intermediate risk aged 55-74 years, and at high risk aged 50-74 years) would be associated with 6% more breast cancer deaths averted than B40-74 (7.2 vs 6.8 per 1000 women) and 13% fewer false-positive recalls (1190 vs 1365 per 1000 women). Results were consistent across the 2 CISNET models, and the relative difference in breast cancer deaths averted between B40-74 and risk-based screening strategies was more pronounced than for life-years gained.

**Conclusions and Relevance:**

In this decision analytical modeling study of breast cancer screening, population risk-based screening using 5-year invasive breast cancer risk was associated with similar or greater benefits than age-based screening as well as reduced false-positive recalls. As personalized medicine advances, risk-based screening is poised to become a cornerstone of breast cancer prevention, offering a more nuanced and tailored approach to patient care.

## Key Points

**Question:**

Are breast cancer screening strategies personalized by a woman’s 5-year absolute risk associated with improved effectiveness and reduced harms compared with traditional age-based screening approaches?

**Findings:**

In this decision analytical model using collaborative simulation modeling of 2 validated CISNET models, 9 risk-based screening strategies were associated with a comparable or greater number of averted breast cancer deaths than biennial age-based screening from ages 40 to 74 years (B40-74) and reduced false-positive recalls by 8% to 23%. For example, compared with B40-74, a risk-based strategy would be associated with 6% more averted breast cancer deaths and 13% fewer false-positive recalls per 1000 women.

**Meaning:**

This study suggests that risk-based mammography screening using 5-year absolute risk may be associated with improved breast cancer mortality outcomes and reduced harms compared with age-based screening approaches.

## Teaser

This decision analytical model study used collaborative simulation modeling to compare the effectiveness and harms associated with mammography screening strategies that vary by 5-year risk of invasive breast cancer vs age-based strategies.

## Introduction

Early detection of breast cancer through screening mammography has contributed to the observed reduction in breast cancer mortality in the US over the past 4 decades.^1,2^ Although specific screening guidelines vary across major medical organizations, they generally endorse recommendations for screening periodicity, with age as the primary criterion for starting and stopping screening (age-based screening). Risk-based screening has been used primarily for women at very high risk who are eligible for supplemental breast magnetic resonance imaging screening, with guidelines focusing on those with high-penetrance genetic variants or other significant risk factors associated with a lifetime breast cancer risk exceeding 20%.^3^ However, given the variation in breast cancer risk among the general population and the availability of validated tools to assess their risk in clinical practice,^4,5,6,7^ there is an opportunity to design more tailored screening programs that will maximize the benefit by screening women at high risk and reduce harms in women at low risk. Thus, by targeting women outside the highest-risk groups (eg, breast cancer survivors, variant carriers, those with prior radiotherapy exposure, or high-risk benign lesions), risk-based screening could be associated with improved screening effectiveness and reduced associated harms. For example, women with high breast density are 3 to 4 times more likely to develop breast cancer compared with those with low breast density, yet not all women with high breast density fall into the highest-risk group.^8,9,10^ Using screening strategies that account for changes in absolute risk over time could enhance effectiveness over current recommendations by ensuring women at higher than average risk receive tailored screening, while those at low risk are less frequently screened.^11^

Several ongoing prospective trials are directly comparing age-based and risk-based breast cancer screening strategies and are expected to report outcomes shortly. However, these studies may have limited power to assess long-term outcomes such as breast cancer mortality and typically evaluate only a few specific risk-stratified screening combinations.^12,13^ In the absence of trial data, simulation modeling offers a rigorous and cost-effective approach, functioning as a virtual laboratory that reduces time requirements.^14^

Consideration of individual breast cancer risk requires that clinicians and women have access to accurate, well-calibrated risk assessment tools that can be used periodically to reassess and update risk over time. To this end, several breast cancer risk assessment models, including the Gail model and the Breast Cancer Surveillance Consortium (BCSC) risk calculator, have been successfully developed and validated.^4,5^ For example, the BCSC risk calculator has been available for over 17 years and was developed using data from 1.5 million US women aged 35 to 79 years who underwent screening between 2000 and 2017.^5^

In this study, 2 independently developed and validated breast cancer simulation models from the National Cancer Institute–funded Cancer Intervention and Surveillance Modeling Network (CISNET)^2,14,15,16^ were used to compare the effectiveness of various risk-based screening strategies with the most commonly recommended age-based screening recommendations. The results aim to inform clinical guidelines and support shared decision-making regarding mammography strategies for women with different risk factors across the range of screening-eligible ages.

## Methods

The decision analytical modeling study used publicly available and deidentified data and was deemed exempt or not human participants research by the institutional review boards at Erasmus University and University of Wisconsin–Madison. Two CISNET models were used in this study^14^: model E (Erasmus University)^17^ and model W (University of Wisconsin–Madison).^18^ The details of model inputs, assumptions, and structure are extensively described on the CISNET website^19^ and in prior publications.^14,17,18,20,21,22^ Modeling analyses were conducted from April 2023 to April 2025. The 2022 Consolidated Health Economic Evaluation Reporting Standards (CHEERS) reporting guideline for decision modeling was followed in this study.^23^

### Model Descriptions

CISNET breast cancer models have been independently developed and validated to address breast cancer prevention and control problems. These include estimating the association of treatment and screening advancements over the past 4 decades with subtype-specific breast cancer mortality.^2,24^ In addition, the models have provided data to inform US Preventive Services Task Force (USPSTF) breast cancer guidelines in 2009, 2016, and 2024.^15,16,22^

Although the 2 CISNET models included in this analysis share common data inputs (eAppendix 1, eTable 1, and eTable 2 in Supplement 1), differences in their structures and assumptions result in a range of likely outcomes, providing a valuable measure of model structural uncertainty regarding inherently unobservable phenomena, such as preclinical parameters of breast cancer natural history and incidence in the absence of screening.^2^ In previous works, the models have been successfully validated against US incidence and mortality trends as well as the UK Age screening trial.^2,25^

#### Population

A single cohort of US women born in 1980 was simulated until death. Consistent with population-based screening guidelines,^26^ a range of breast cancer risk levels was modeled, including women with factors associated with an increased risk of breast cancer, such as a family history of breast cancer and a high breast density. However, women at the highest risk, such as those with a genetic variant or syndrome (eg, *BRCA1/2*), a history of high-dose radiotherapy to the chest at a young age, and/or previous breast cancer, were not included.

#### Model Input Parameters

A common set of large, national data sources (eAppendix 1 and eTable 1 in Supplement 1) was used to inform model input parameters. The assumptions for the models are the same as those described in the analysis conducted for the 2024 USPSTF guideline development.^22^ Model W has since been updated to explicitly incorporate the diagnosis of metastatic recurrence and postmetastatic survival.^24^ It was assumed that 100% of modeled women completed all lifetime screening examinations within each modeled screening strategy.

Data from the BCSC were used to inform the sensitivity of digital breast tomosynthesis by breast density, which was classified according to the Breast Imaging Reporting and Data Systems (BI-RADS) categories: almost entirely fatty (category a), scattered fibroglandular density (category b), heterogeneously dense (category c), and extremely dense (category d).^27^ Some women experience a decrease in density category at ages 50 and/or 65 years, based on age-specific prevalence observed in the BCSC. Breast density was assumed to be associated with age-specific screening performance and 5-year breast cancer risk, but not cancer natural history.

#### Absolute 5-Year Risk of Invasive Breast Cancer Diagnosis

The BCSC risk calculator model, version 3, which estimates 5-year absolute risk of invasive cancer, was used for several reasons.^5^ It is recommended by the USPSTF for determining risk for primary prevention.^28^ The model is well calibrated and has been externally validated in 3 different cohorts,^5,29,30,31^ demonstrating the highest area under the receiver operating characteristic curve value among breast cancer risk prediction models when breast density information is included.^32^ The calculator is available for free as an app and online.^33^ Table 1 provides sample patient profiles with 5-year risk estimates calculated using the BCSC risk calculator.

**Table 1.** Example Profiles of Women in Different Risk Groups

No.	Age, y	Race or ethnicity	Family history of breast cancer	Benign biopsy result	Breast density category^a^	Menopausal status	BMI	Age range at first live birth, y	Estimated 5-y risk, %	Risk group across all age groups	Risk group within the 5-y age group
1	40	Hispanic	None	No	a	Premenopausal	19-25	20-24	0.10	Low	Low
2	40	Black	None	No	a	Premenopausal	19-25	20-24	0.13	Low	Low
3	40	White	None	No	a	Premenopausal	19-25	20-24	0.13	Low	Low
4	40	Black	Second degree only	No	a	Premenopausal	19-25	20-24	0.18	Low	Low
5	40	White	None	No	d	Premenopausal	19-25	20-24	0.70	Low	Average
6	40	White	Second degree only	No	d	Premenopausal	19-25	20-24	0.95	Average	Intermediate
7	40	White	≥2 First degree	No	d	Premenopausal	19-25	20-24	2.01	Intermediate	High
8	55	White	None	No	a	Postmenopausal	19-25	20-24	0.39	Low	Low
9	55	White	None	No	d	Postmenopausal	19-25	20-24	1.37	Average	Average
10	55	Black	Second degree only	No	d	Postmenopausal	19-25	20-24	1.57	Average	Average
11	55	White	Second degree only	No	d	Postmenopausal	19-25	20-24	1.61	Average	Average
12	55	White	Second degree only	No	d	Postmenopausal	25-29	20-24	2.13	Intermediate	Intermediate
13	50	Black	≥2 First degree	No	d	Postmenopausal	19-25	20-24	2.24	Intermediate	High
14	55	White	≥2 First degree	No	d	Postmenopausal	19-25	20-24	2.77	High	High
15	55	White	≥2 First degree	No	c	Postmenopausal	19-25	20-24	2.31	Intermediate	High
16	55	White	≥2 First degree	No	b	Postmenopausal	19-25	20-24	1.50	Average	Average
17	55	White	≥2 First degree	No	a	Postmenopausal	19-25	20-24	0.79	Low	Low
18	60	Hispanic	≥2 First degree	No	d	Postmenopausal	19-25	20-24	2.36	Intermediate	Intermediate
19	60	Black	≥2 First degree	No	d	Postmenopausal	19-25	20-24	2.75	High	High
20	60	White	≥2 First degree	No	d	Postmenopausal	19-25	20-24	2.98	High	High

Abbreviation: BMI, body mass index (calculated as weight in kilograms divided by height in meters squared).

^a^ Breast density definition is based on the Breast Imaging Reporting and Data Systems categories: almost entirely fatty (a), scattered fibroglandular density (b), heterogeneously dense (c), and extremely dense (d). The risk factors and the risk estimates are sourced from Breast Cancer Surveillance Consortium risk calculator, which is available online.^33^

#### Simulated Screening Strategies

A no-screening scenario was simulated as the reference case, along with 3 commonly recommended age-based screening scenarios: biennial screening for women aged 40 to 74 years (B40-74), as recommended by the USPSTF in their 2024 guidelines^26^; biennial screening for women aged 50 to 74 years (B50-74), as recommended by the American College of Physicians^34^; and annual screening for women aged 40 to 74 years (A40-74), serving as a proxy for guidelines from the American College of Radiology^35^ and the National Comprehensive Cancer Network,^36^ both of which recommend annual screening beginning at 40 years of age (Table 2). To implement risk-based screening, the BCSC risk calculator was applied to 1 455 493 women aged 35 to 79 years who received mammography at BCSC facilities between 2000 and 2017 and had complete risk factor data.^5^ The women were then sorted by their 5-year risk from lowest to highest, within each 5-year age group (eg, 35-39 years, 40-44 years, and 45-49 years) and breast density category. Each simulated woman was assigned a 5-year risk at 40 years of age, which was updated every 5 years throughout her lifetime during the simulation. In both models, the 5-year absolute risk was associated only with cancer initiation and was not associated with other components of cancer natural history, including cancer subtype or duration of the preclinical detectable phase, or postdiagnosis treatment and survival. To demonstrate that the extended models accurately reproduced absolute risks, 2 internal validation experiments were conducted (eAppendix 3, eTable 7, and eFigure 1 in Supplement 1).

**Table 2.** Summary of the Categories of Simulated Mammography Screening Scenarios^a^

Assignment strategy and age groups screened, y	Threshold 5-y absolute risk values for risk groups, %				No. of scenarios
	Low risk	Average risk	Intermediate risk	High risk	
Reference scenario: no screening					
NA	NA	NA	NA	NA	1
Category A: age-based screening scenarios					
Biennial screening					
40-74	NA	NA	NA	NA	3
50-74	NA	NA	NA	NA	
Annual screening					
40-74	NA	NA	NA	NA	
Category B: risk-based screening using overall thresholds starting at age 40 y					
40-74	≤0.80	0.81-1.79	1.80-2.41	≥2.42	9
Category C: risk-based screening using age-specific thresholds starting at age 40 y					
40-44	≤0.44	0.45-0.82	0.83-1.04	≥1.05	24
45-49	≤0.64	0.65-1.18	1.19-1.46	≥1.47	
50-54	≤0.83	0.84-1.47	1.48-1.79	≥1.80	
55-59	≤0.98	0.99-1.69	1.70-2.16	≥2.17	
60-64	≤1.22	1.23-2.03	2.04-2.65	≥2.66	
65-69	≤1.39	1.40-2.32	2.33-2.99	≥3.00	
70-74	≤1.53	1.54-2.47	2.48-3.26	≥3.27	
Category D: risk-based screening using overall thresholds starting at age 50 y					
50-74	≤0.80	0.81-1.79	1.80-2.41	≥2.42	9
Category E: risk-based screening using age-specific thresholds starting at age 50 y					
50-54	≤0.83	0.84-1.47	1.48-1.79	≥1.80	5
55-59	≤0.98	0.99-1.69	1.70-2.16	≥2.17	
60-64	≤1.22	1.23-2.03	2.04-2.65	≥2.66	
65-69	≤1.39	1.40-2.32	2.33-2.99	≥3.00	
70-74	≤1.53	1.54-2.47	2.48-3.26	≥3.27	

Abbreviation: NA, not applicable.

^a^ Each scenario in the risk-based screening strategies had a unique screening interval policy (eg, biennial screening for women at low risk and annual screening for women at average, intermediate, and high risk; biennial screening for women at low and average risk and annual screening for women at intermediate and high risk). For the full description of scenarios investigated see eTables 3-6 in Supplement 1.

A total of 47 risk-based screening strategies were simulated (Table 2). Screening strategies varied by the age of initial risk and density assessment (40 or 50 years) and whether overall population or age-specific risk thresholds were used to determine the threshold values for risk categorization based on the 5-year risk. In risk-based screening strategies using overall thresholds (categories B and D in Table 2), 5-year risk groups were based on quartiles across all ages: the 25th percentile (≤0.80%) for low risk, 25th to 75th percentile for average risk (0.81%-1.79%), 75th to 90th percentile for intermediate risk (1.80%-2.41%), and 90th percentile and above for high risk (≥2.42%). These 5-year risk threshold values were applied to women of all ages. In contrast, risk-based screening strategies using age-specific thresholds (categories C and E in Table 2) categorized risk groups separately within each 5-year age group, using the same percentile definitions (eg, lowest 25% within a given 5-year age group were classified as low risk and those in the 25%-75% range as average risk) but based on the age-specific distribution observed in the BCSC.

In both risk-based screening strategies, a woman’s 5-year risk was updated throughout her lifetime. The key distinction between these strategies lies in how risk thresholds were determined for categorizing women into different risk groups. In risk-based screening using overall thresholds, the risk levels used to classify women as being at low, average, intermediate, or high risk remained constant across all ages. In contrast, risk-based screening using age-specific thresholds adjusted these risk levels within each 5-year age group. Furthermore, risk-based screening with age-specific thresholds allowed screening intervals to be adjusted for each 5-year age group, providing a more flexible and dynamic screening approach.

Strategies B/C and D/E additionally assumed that all women first underwent mammography at ages 40 or 50 years, respectively, during which their breast density category was determined. The full list of risk-based screening strategies is provided in eAppendix 2 and eTables 3 to 6 in Supplement 1. Whenever a woman transitioned to a new screening strategy due to the changes in her 5-year risk over time, the new strategy commenced only after the last round of screening under the previous strategy had been completed.

### Statistical Analysis

Each model simulated the lifetime outcomes of 50 screening strategies (3 age based and 47 risk based) and a no-screening scenario. The primary outcomes were lifetime breast cancer deaths averted and false-positive recalls per 1000 women screened. Secondary outcomes included life-years saved with screening, number of mammography screenings, and benign biopsies per 1000 women screened. Results are reported using the mean and range across the 2 models.

Efficiency and near-efficiency frontiers plotted the sequence of the most efficient strategies, which was associated with the largest incremental benefits of screening relative to screening burden (eg, deaths averted per false-positive recall). A strategy was labeled *near efficient* if its associated outcome was within 5% of the value obtained by an efficient strategy.^22^ Data analysis for the results reported in this study was performed with Excel version 2505 (Microsoft Corporation), R Studio, version 4 (Posit Team), and SAS, version 9.4 (SAS Institute Inc).

## Results

### Primary Outcomes

The mean model results estimated that strategy B40-74, compared with no screening, would be associated with 6.8 averted breast cancer deaths per 1000 women (range, 5.5-8.2 per 1000 women) and 1365 false-positive recalls per 1000 women (range, 1355-1374 per 1000 women) (Table 3; eAppendix 4 and eTable 8 in Supplement 1). Nine risk-based screening strategies were associated with a comparable or greater number of deaths averted (range across strategies for mean model estimates, 6.8-7.5 per 1000 women) than B40-74 (6.8 per 1000 women) as well as reduced false-positive recalls by 8% to 23%. For example, compared with B40-74, the risk-based strategy using overall risk thresholds for women aged 40 to 74 years (strategy B2 in Table 3), which involved no screening for women at low risk (ie, 5-year risk, ≤0.80%), biennial screening for women at average risk (ie, 5-year risk, 0.81%-1.79%), and annual screening for women at intermediate and high risk (ie, 5-year risk, 1.80%-2.41% and ≥2.42%, respectively), would be associated with 6% more averted breast cancer deaths (7.2 per 1000 women [range, 6.0-8.4 per 1000 women] vs 6.8 per 1000 women [range, 5.5-8.2 per 1000 women]) as well as 8% fewer false-positive recalls (1257 per 1000 women [range, 1256-1258 per 1000 women] vs 1365 per 1000 women [range, 1355-1374 per 1000 women]). Similarly, compared with B40-74, biennial screening for women at low risk and annual screening for women at average, intermediate, and high risk between 50 and 74 years of age (strategy D9 in Table 3) would be associated with 10% more averted breast cancer deaths (7.5 per 1000 women [range, 6.2-8.7 per 1000 women] vs 6.8 per 1000 women [range, 5.5-8.2 per 1000 women]), as well as 10% fewer false-positive recalls (1232 per 1000 women [range, 1221-1242 per 1000 women] vs 1365 per 1000 women [range, 1355-1374 per 1000 women]). Moreover, compared with B40-74, a risk-based screening strategy using age-specific thresholds (strategy C4 in Table 3) using a combination of biennial screening (for women at low risk aged 55-74 years, at average risk aged 50-59 years, at intermediate risk aged 45-54 years, and at high risk aged 40-49 years) and annual screening (for women at average risk aged 60-74 years, at intermediate risk aged 55-74 years, and at high risk aged 50-74 years) would be associated with 6% more averted breast cancer deaths (7.2 per 1000 women [range, 5.9-8.4 per 1000 women] vs 6.8 per 1000 women [range, 5.5-8.2 per 1000 women]) as well as 13% fewer false-positive recalls (1190 per 1000 women [range, 1184-1196 per 1000 women] vs 1365 per 1000 women [range, 1355-1374 per 1000 women]). Across all strategies, compared with B40-74, risk-based screening was associated with reduced false-positive recalls by 8% to 23% (1365 per 1000 women [range, 1355-1374 per 1000 women] vs 1050 per 1000 women [range, 1047-1053 per 1000 women] to 1257 per 1000 women [range, 1256-1258 per 1000 women]) (Table 3).

**Table 3.** Selected Risk-Based Screening Strategies Associated With a Comparable or Greater Reduction in Breast Cancer Deaths and Reduced False-Positive Recalls Compared With Biennial Screening Between 40 and 74 Years of Age According to the Mean Outcome Per 1000 Women^a^

Strategy	Age at risk assessment, y	Screening strategy by risk group				No. of mammography screenings (range)	No. of false-positive recalls per 1000 women (range)	No. of benign biopsies per 1000 women (range)	Breast cancer deaths averted per 1000 women (range)	No. of life-years gained per 1000 women (range)
		Low risk	Average risk	Intermediate risk	High risk					
B40-74	NA	B	B	B	B	15 960 (15 835-16 086)	1365 (1355-1374)	200 (198-201)	6.8 (5.5-8.2)	127 (105-150)
B50-74	NA	B	B	B	B	11 088 (10 983-11 194)	864 (856-872)	135 (133-136)	5.7 (4.5-6.9)	95 (77-112)
A40-74	NA	A	A	A	A	30 877 (30 591-31 162)	2074 (2057-2091)	285 (283-288)	9.2 (7.7-10.6)	176 (151-201)
B2	40	NS	B	A	A	16 434 (16 410-16 457)	1257 (1256-1258)	186 (185-186)	7.2 (6.0-8.4)	128 (109-148)
C1	40	NS if age <55 y; B if age ≥55 y	NS if age <50 y; B if age 50-59 y; A if age ≥60 y	NS if age <45 y; B if age 45-49 y; A if age ≥50 y	B if age <50 y; A if age ≥50 y	17 386 (17 262-17 510)	1203 (1195-1211)	177 (176-178)	7.2 (6.0-8.4)	126 (106-145)
C2	40	NS if age <55 y; B if age ≥55 y	NS if age <50 y; B if age 50-59 y; A if age ≥60 y	NS if age <45 y; B if age 45-54 y; A if age ≥55 y	B if age <55 y; A if age ≥55 y	16 911 (16 857-16 965)	1180 (1177-1183)	174 (174-175)	7.1 (5.9-8.3)	123 (104-141)
C3	40	NS if age <55 y; B if age ≥55 y	NS if age <50 y; B if age 50-59 y; A if age ≥60 y	B if age <60 y; A if age ≥60 y	B if age <55 y; A if age ≥55 y	16 805 (16 721-16 888)	1186 (1181-1192)	175 (174-176)	7.1 (5.9-8.3)	123 (105-141)
C4	40	NS if age <55 y; B if age ≥55 y	NS if age <50 y; B if age 50-59 y; A if age ≥60 y	NS if age <45 y; B if age 45-54 y; A if age ≥55 y	B if age <50 y; A if age ≥50 y	17 119 (17 023-17 215)	1190 (1184-1196)	176 (175-176)	7.2 (5.9-8.4)	124 (105-143)
D9	50	B	A	A	A	20 224 (20 051-20 398)	1232 (1221-1242)	180 (179-182)	7.5 (6.2-8.7)	126 (107-145)
E1	50	NS if age <55 y; B if age ≥55 y	B if age 50-59 y; A if age ≥60 y	A	A	16 347 (16 172-16 522)	1072 (1061-1083)	161 (159-162)	6.9 (5.7-8.1)	115 (97-133)
E3	50	NS if age <55 y; B if age ≥55 y	B if age 50-59 y; A if age ≥60 y	B if age 50-54 y; A if age ≥55 y	B if age 50-54 y; A if age ≥55 y	15 836 (15 787-15 886)	1050 (1047-1053)	158 (158-159)	6.8 (5.6-8.0)	112 (96-129)
E4	50	NS if age <55 y; B if age ≥55 y	B if age 50-59 y; A if age ≥60 y	A	A	17 718 (17 529-17 906)	1109 (1098-1120)	165 (163-166)	7.1 (5.9-8.3)	117 (99-135)

Abbreviations: A, annual; B, biennial; NA, not applicable; NS, no screening.

^a^ Values in parentheses indicate outcomes from model W (first value [University of Wisconsin–Madison]) and model E (second value [Erasmus University]).

### Efficiency Frontiers

**Figure 1.**
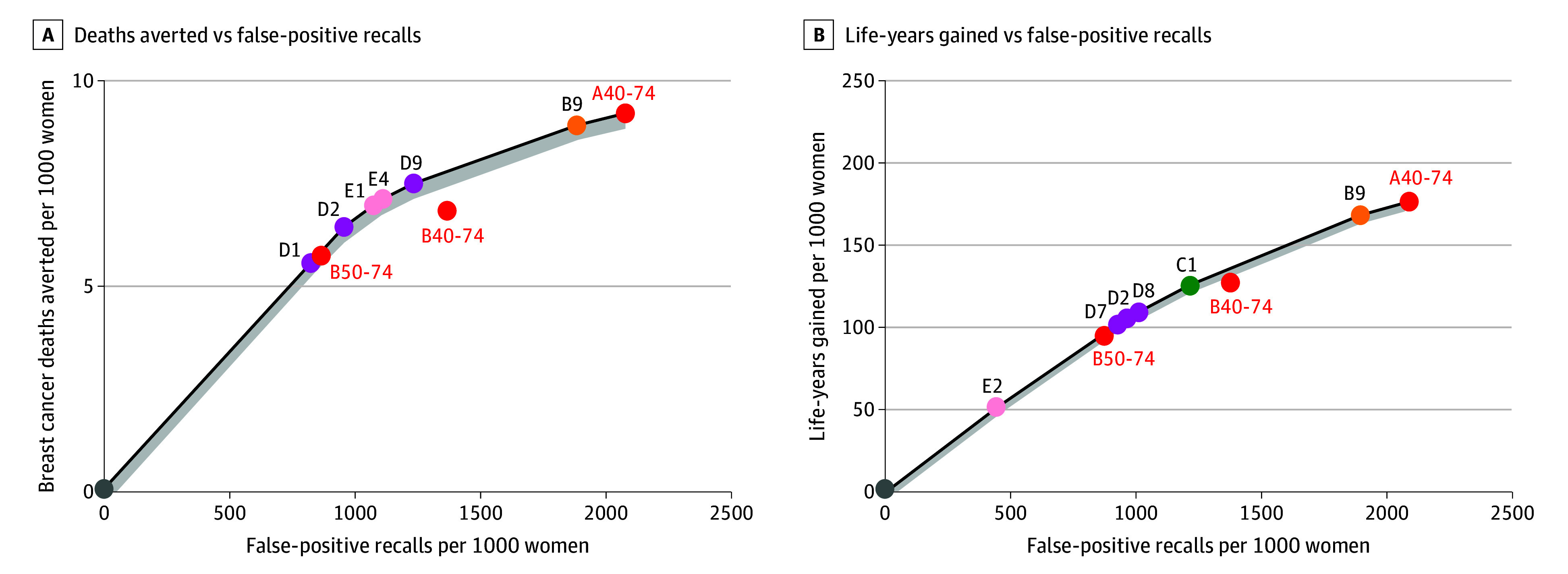
Efficiency Frontiers for the Estimated Lifetime Number of False-Positive Recalls, Breast Cancer Deaths Averted, and Life-Years Gained for a Cohort of 1000 Women by Screening Strategy A, Deaths averted vs false-positive recalls. B, Life-years gained vs false-positive recalls. Outcomes shown are means of the outcomes estimated by model W (University of Wisconsin–Madison) and model E (Erasmus University). Gray shading indicates the area within 5% of the value surrounding efficient strategies, where near-efficient strategies are displayed. B40-74 indicates biennial screening for women aged 40 to 74; B50-74, biennial screening for women aged 50 to 74; A40-74, annual screening for women aged 40 to 74. Details of the risk-based screening strategies that appeared on the efficiency frontier are provided in Table 3.

**Figure 2.**
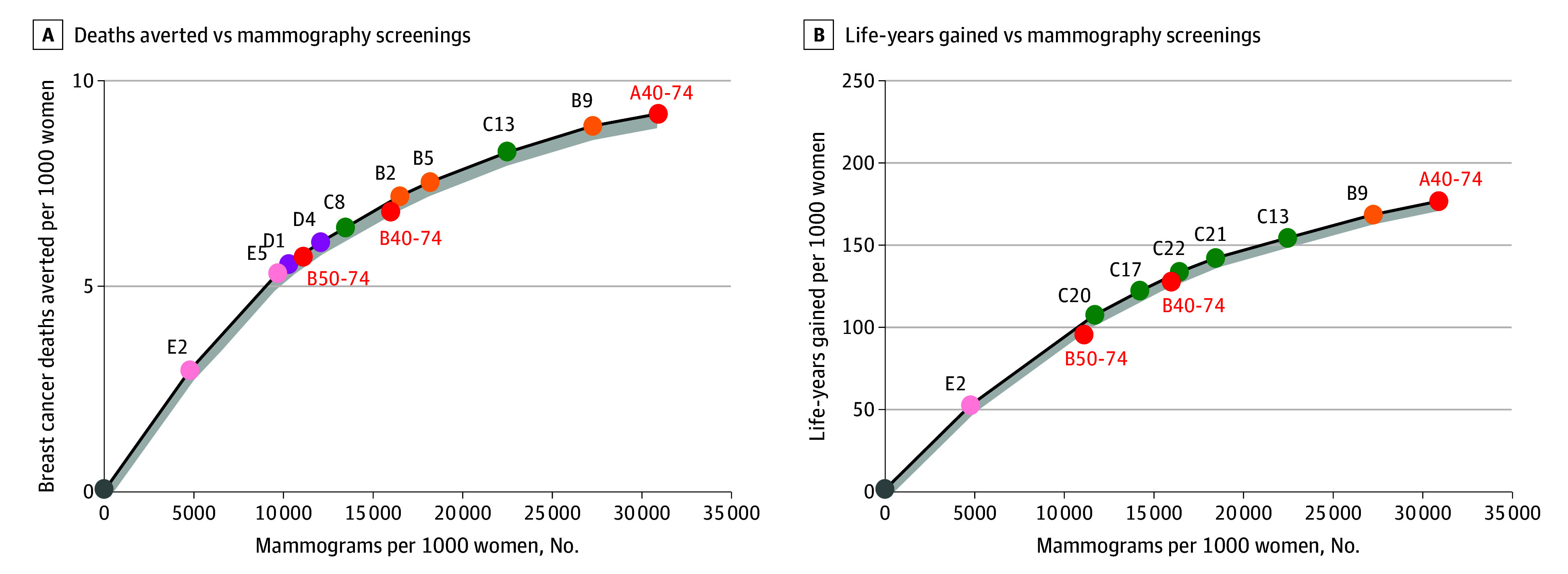
Efficiency Frontiers for the Estimated Lifetime Number of Mammography Screenings, Breast Cancer Deaths Averted, and Life-Years Gained for a Cohort of 1000 Women by Screening Strategy A, Deaths averted vs mammography screenings. B, Life-years gained vs mammography screenings. Outcomes shown are means of the outcomes estimated by model W and model E. Grey shading indicates the area within 5% of the value surrounding efficient strategies, where near-efficient strategies are displayed. B40-74 indicates biennial screening for women aged 40 to 74; B50-74, biennial screening for women aged 50 to 74; A40-74, annual screening for women aged 40 to 74. Details of the risk-based screening strategies that appeared on the efficiency frontier are provided in Table 3.

In general, both models produced similar estimates for risk-based screening compared with age-based screening and identified the same set of strategies on the efficiency frontiers, providing further confidence in the results based on mean model estimates (Figure 1 and Figure 2; eAppendix 4 and eFigures 2-5 in Supplement 1). B40-74 does not appear on the efficiency frontier when the number of false-positive recalls is used as the harm metric (Figure 1). When the number of mammography screenings is used as the harm metric, B40-74 is near the efficiency frontier (Figure 2). Furthermore, the relative difference in life-years gained between B40-74 and risk-based screening strategies is less pronounced than the difference in breast cancer deaths averted (Figure 1 and Figure 2).

The efficiency frontiers predicted by the models did not change substantially when benign biopsy outcomes were used instead of false-positive recalls, or when the percentage reduction in breast cancer mortality was used instead of breast cancer deaths averted (eFigures 6 and 7 in Supplement 1). Although only a few risk-based screening strategies appeared on the efficiency frontier, many were classified as near efficient (eFigures 8 and 9 in Supplement 1).

### Risk-Based Screening Strategies Using Overall Population vs Age-Specific Risk Thresholds

Risk-based screening strategies using overall population thresholds were associated with similar benefit or harm outcomes compared with risk-based screening using age-specific thresholds, although strategies using age-specific thresholds were associated with greater reductions in false positives. Risk-based screening strategies initiating risk assessment at 40 years of age are more likely to appear on the efficiency frontier when life-years gained is used as the benefit metric and the number of mammography screenings is used as the harm metric (Figure 1 and Figure 2; and eAppendix 5 and eTable 9 in Supplement 1). In contrast, risk-based screening strategies initiating risk assessment at 50 years of age (categories D and E in Table 2) are more likely to appear on the efficiency frontier when breast cancer deaths averted is used as the benefit metric and the number of false-positive recalls is used as the harm metric (Figure 1 and Figure 2; eAppendix 5 and eTable 9 in Supplement 1).

## Discussion

To our knowledge, this decision analytical modeling study is the first to consider 5-year absolute invasive breast cancer risk to tailor population screening strategies in the US. The models project that risk-based breast cancer screening strategies could be associated with comparable or greater reductions in deaths from breast cancer compared with biennial screening between 40 and 74 years of age for all women, as well as decreased false-positive recalls and benign biopsies. The study further found that the ranking of the strategies as efficient or near efficient varied depending on the outcome measures used. In general, the relative difference in breast cancer deaths averted between biennial screening between 40 and 74 years of age and risk-based screening strategies was more pronounced than for life-years gained.

All risk-based screening strategies considered in this study accounted for changes in risk as a woman aged, requiring risk estimates every 5 years or whenever a patient chose to reassess her screening pattern. Risk-based strategies using overall risk thresholds consistently appeared on efficiency frontiers as did age-specific risk thresholds, which were overall associated with greater reductions in false positives. These findings suggest that risk-based screening strategies using either approach are associated with improved benefit or harm outcomes relative to purely age-based screening strategies.

Other studies have compared risk-based and age-based screening outside the US.^11,37,38,39,40^ For example, a study focusing on Germany found that risk-based strategies provided comparable or greater benefits for women aged 40 to 49 years compared with those aged 50 to 69 years, for whom screening is typically recommended in that country.^37^ Although our study used a different study design, our findings align with the conclusion that some women at high risk aged 40 to 49 years may benefit more from screening than older women at low risk.

Risk-based screening represents a paradigm shift in breast cancer early detection. By offering more intensive screening to women at high risk and less intensive screening to women at low risk, these strategies may be associated with similar benefits and reduced screening-associated burden.^41^ For instance, reducing unnecessary mammography and associated diagnostic workup for women at low risk lowers health care costs and decreases patient burden.

### Limitations

Our study has several limitations. First, although ongoing trials are working to define risk percentile–based cutoffs to categorize women into low-, average-, intermediate-, and high-risk groups, universally accepted thresholds are lacking; therefore, we used fixed cutoffs.^13^ Second, this study evaluated risk-based screening using the BCSC risk calculator, which does not incorporate genetic risk factors such as polygenic risk scores. These are being integrated into risk-based screening approaches and evaluated in the ongoing trials.^12,13^ Prior CISNET modeling work has demonstrated the feasibility of incorporating polygenic risk scores to guide screening strategies.^42^ Furthermore, because the BCSC calculator was developed in women with varying screening histories, its absolute risk estimates inherently reflect that context, a limitation common to all existing invasive breast cancer risk models. The actionable advanced cancer risk model generates risk according to screening interval and future work will evaluate this model.^43^ This study assumed that implementing risk-based screening would not add administrative burden or cost, although, in practice, such approaches may involve greater complexity. However, breast cancer risk assessment tools are now available as integrated components of electronic medical records and mammography reporting systems, and as a result the mammography encounter offers an important opportunity for efficient risk-based assessment in screening.^44^ Although this study assumed the absolute risk is associated only with the risk of developing breast cancer, it is likely to also be associated with cancer subtypes as there is a lack of evidence for this association. In addition, while we conducted internal validation experiments to confirm that the BCSC risk calculator’s estimates were correctly incorporated into the models, we could not conduct an external validation as no dataset currently provides the necessary information to externally validate absolute risk-based screening. Furthermore, this study’s assumption that risk is updated every 5 years does not reflect clinical practice, where risk may be reassessed more frequently (eg, biennially). This likely leads to an underestimation of the benefits associated with risk-based screening.

## Conclusions

This decision analytical modeling study of breast cancer screening highlighted the potential of 5-year risk-based breast cancer screening to improve clinical outcomes and resource use at the population level. By shifting the focus from uniform population-wide recommendations based on age alone to individualized risk-based strategies, these approaches were projected to maintain or improve mortality benefits while reducing the burden of screening harms. As personalized medicine advances, risk-based screening is poised to become a cornerstone of breast cancer prevention, offering a more nuanced and tailored approach to patient care.

## Notes

Supplement 1. **eAppendix 1.** Details of the CISNET Models Used in this Study **eTable 1.** Summary of Common Inputs Used by the Models **eTable 2.** Summary of Key Model Features **eAppendix 2.** List of Risk-Based Screening Strategies **eTable 3.** List of Type B Risk-Based Screening Strategies **eTable 4.** List of Type C Risk-Based Screening Strategies **eTable 5.** List of Type D Risk-Based Screening Strategies **eTable 6.** List of Type E Risk-Based Screening Strategies **eAppendix 3.** Internal Validation of the Models **eTable 7.** Comparison of Key Breast Cancer Outcomes Produced by the CISNET Models With and Without the Incorporation of Absolute Risk Under Different Screening Strategies **eFigure 1.** Comparison of Model-Projected Relative Risks and BCSC Risk Calculator Estimates Across Density Categories for Women Aged 40–44 Under no Screening Strategy **eAppendix 4.** Additional Numerical Results **eTable 8.** Estimated Outcomes Associated With Risk-Based Screening Strategies Compared to Biennial Screening for Ages 40-74 and 50-74 and Annual Screening for Ages 40-74 According to the Average Model Outcome **eFigure 2.** Efficiency Frontiers for the Estimated Lifetime Number of False-Positive Mammograms and Breast Cancer Deaths Averted for a Cohort of 1000 Women According to Model and Screening Strategy **eFigure 3.** Efficiency Frontiers for the Estimated Lifetime Number of False Positive Mammograms and Life-Years Gained According to Model and Screening Strategy for a Cohort of 1000 Women **eFigure 4.** Efficiency Frontiers for the Estimated Lifetime Number of Mammograms and Breast Cancer Deaths Averted for a Cohort of 1000 Women According to Model and Screening Strategy **eFigure 5.** Efficiency Frontiers for the Estimated Lifetime Number of Mammograms and Life-Years Gained According to Model and Screening Strategy for a Cohort of 1000 Women **eFigure 6.** Efficiency Frontiers for the Estimated Lifetime Number of False-Positive Mammograms and Percent Reduction in Breast Cancer Mortality for a Cohort of 1000 Women by Screening Strategy **eFigure 7.** Efficiency Frontiers for the Estimated Lifetime Number of Benign Biopsies, Life-Years Gained, and Breast Cancer Deaths Averted for a Cohort of 1000 Women by Screening Strategy **eFigure 8.** Efficiency Frontiers Displaying all Policies for the Estimated Lifetime Number of False-Positive Mammograms, Life-Years Gained, and Breast Cancer Deaths Averted for a Cohort of 1000 Women by Screening Strategy **eFigure 9.** Efficiency Frontiers Displaying all Policies for the Estimated Lifetime Number of Mammograms, Life-Years Gained, and Breast Cancer Deaths Averted for a Cohort of 1000 Women by Screening Strategy **eAppendix 5.** Impact of Initiation of Screening at Age 40 on the Projected Outcomes **eTable 9.** Distribution of Risk Categories Within Each Age Group and Distribution of Age Groups Within Each Risk Category for Strategies B2 and D9
eReferences

Supplement 2.
Data Sharing Statement
