# Supplementary material for: Five-Year Absolute Risk–Based and Age-Based Breast Cancer Screening in the US
Source: JAMA Netw Open. 2026 Jan 20;9(1):e2552944. doi: 10.1001/jamanetworkopen.2025.52944 (PMC12820738; doi:10.1001/jamanetworkopen.2025.52944)
Supplement: Supplement 1. — eAppendix 1. Details of the CISNET Models Used in this Study eTable 1. Summary of Common Inputs Used by the Models eTable 2. Summary of Key Model Features eAppendix 2. List of Risk-Based Screening Strategies eTable 3. List of Type B Risk-Based Screening Strategies eTable 4. List of Type C Risk-Based Screening Strategies eTable 5. List of Type D Risk-Based Screening Strategies eTable 6. List of Type E Risk-Based Screening Strategies eAppendix 3. Internal Validation of the Models eTable 7. Comparison of Key Breast Cancer Outcomes Produced by the CISNET Models With and Without the Incorporation of Absolute Risk Under Different Screening Strategies eFigure 1. Comparison of Model-Projected Relative Risks and BCSC Risk Calculator Estimates Across Density Categories for Women Aged 40–44 Under no Screening Strategy eAppendix 4. Additional Numerical Results eTable 8. Estimated Outcomes Associated With Risk-Based Screening Strategies Compared to Biennial Screening for Ages 40-74 and 50-74 and Annual Screening for Ages 40-74 According to the Average Model Outcome eFigure 2. Efficiency Frontiers for the Estimated Lifetime Number of False-Positive Mammograms and Breast Cancer Deaths Averted for a Cohort of 1000 Women According to Model and Screening Strategy eFigure 3. Efficiency Frontiers for the Estimated Lifetime Number of False Positive Mammograms and Life-Years Gained According to Model and Screening Strategy for a Cohort of 1000 Women eFigure 4. Efficiency Frontiers for the Estimated Lifetime Number of Mammograms and Breast Cancer Deaths Averted for a Cohort of 1000 Women According to Model and Screening Strategy eFigure 5. Efficiency Frontiers for the Estimated Lifetime Number of Mammograms and Life-Years Gained According to Model and Screening Strategy for a Cohort of 1000 Women eFigure 6. Efficiency Frontiers for the Estimated Lifetime Number of False-Positive Mammograms and Percent Reduction in Breast Cancer Mortality for a Cohort of 1000 Women by Screening Strategy eF [file jamanetwopen-e2552944-s001.pdf]

Supplementary Online Content

Alagoz O, Lu Y, Gil Quessep E, et al. Five-year absolute risk–based and age-based breast cancer screening in the US. *JAMA Netw Open.* 2026;9(1):e2552944. doi:10.1001/jamanetworkopen.2025.52944

eAppendix 1. Details of the CISNET Models Used in this Study
eTable 1. Summary of Common Inputs Used by the Models
eTable 2. Summary of Key Model Features
eAppendix 2. List of Risk-Based Screening Strategies
eTable 3. List of Type B Risk-Based Screening Strategies
eTable 4. List of Type C Risk-Based Screening Strategies
eTable 5. List of Type D Risk-Based Screening Strategies
eTable 6. List of Type E Risk-Based Screening Strategies
eAppendix 3. Internal Validation of the Models
eTable 7. Comparison of Key Breast Cancer Outcomes Produced by the CISNET Models With and Without the Incorporation of Absolute Risk Under Different Screening Strategies
eFigure 1. Comparison of Model-Projected Relative Risks and BCSC Risk Calculator Estimates Across Density Categories for Women Aged 40–44 Under no Screening Strategy
eAppendix 4. Additional Numerical Results
eTable 8. Estimated Outcomes Associated With Risk-Based Screening Strategies Compared to Biennial Screening for Ages 40-74 and 50-74 and Annual Screening for Ages 40-74 According to the Average Model Outcome
eFigure 2. Efficiency Frontiers for the Estimated Lifetime Number of False-Positive Mammograms and Breast Cancer Deaths Averted for a Cohort of 1000 Women According to Model and Screening Strategy
eFigure 3. Efficiency Frontiers for the Estimated Lifetime Number of False-Positive Mammograms and Life-Years Gained According to Model and Screening Strategy for a Cohort of 1000 Women
eFigure 4. Efficiency Frontiers for the Estimated Lifetime Number of Mammograms and Breast Cancer Deaths Averted for a Cohort of 1000 Women According to Model and Screening Strategy
eFigure 5. Efficiency Frontiers for the Estimated Lifetime Number of Mammograms and Life-Years Gained According to Model and Screening Strategy for a Cohort of 1000 Women
eFigure 6. Efficiency Frontiers for the Estimated Lifetime Number of False-Positive Mammograms and Percent Reduction in Breast Cancer Mortality for a Cohort of 1000 Women by Screening Strategy
eFigure 7. Efficiency Frontiers for the Estimated Lifetime Number of Benign Biopsies, Life-Years Gained, and Breast Cancer Deaths Averted for a Cohort of 1000 Women by Screening Strategy

© 2026 Alagoz O et al. *JAMA Network Open.*

eFigure 8. Efficiency Frontiers Displaying all Policies for the Estimated Lifetime Number of False-Positive Mammograms, Life-Years Gained, and Breast Cancer Deaths Averted for a Cohort of 1000 Women by Screening Strategy
eFigure 9. Efficiency Frontiers Displaying all Policies for the Estimated Lifetime Number of Mammograms, Life-Years Gained, and Breast Cancer Deaths Averted for a Cohort of 1000 Women by Screening Strategy
eAppendix 5. Impact of Initiation of Screening at Age 40 on the Projected Outcomes
eTable 9. Distribution of Risk Categories Within Each Age Group and Distribution of Age Groups Within Each Risk Category for Strategies B2 and D9
eReferences.

This supplementary material has been provided by the authors to give readers additional information about their work.

© 2026 Alagoz O et al. *JAMA Network Open.*

eAppendix 1. Details of the CISNET Models Used in this Study

This study included three National Cancer Institute (NCI)-funded Cancer Intervention and Surveillance Modeling Network (CISNET) breast cancer models. While the models utilize common inputs including breast cancer incidence and survival in the absence of screening, screening and treatment dissemination over time, performance of screening mammography, treatment effectiveness, they make different assumptions on the natural history of breast cancer. The models calibrate the input parameters governing the natural history of breast cancer and match observed U.S. population incidence and mortality over time as reported by NCI's Surveillance, Epidemiology, and End Results database. **eTable 1** lists the common inputs used by the models and **eTable 2** compares the key assumptions used in the models.

© 2026 Alagoz O et al. *JAMA Network Open.*

eTable 1. Summary of Common Inputs Used by the Models (Adopted from previous publications[1, 2])

Input	Description	Source
Breast cancer incidence in the absence of screening	Age-period-cohort model with SEER breast cancer incidence, removing the period effect of mammography	Holford et al. (2005)[3], Gangnon et al. (2015)[4]
Breast density	Prevalence of breast density (BI-RADS A, B, C, D) by age groups (40-44, 45-49, 50-64, 65-74, 75-89)	BCSC[5]
Other-cause mortality	Age- and cohort-specific mortality rates from deaths other than breast cancer	Gangnon et al. (2018)[6]
Survival in the absence of screening and treatment	25-year breast cancer survival before systematic treatment by joint ER/HER2 status, age group, AJCC/SEER stage or tumor size	Plevritis et al. (2018)[7], Caswell-Jin et al. (2024)[8]
Breast cancer stage distribution	Stage distributions by mode of detection, age group (40-44, 45-49, 50-64, 65-74, 75-89), screening round (first, subsequent), screening interval and breast density (A, B, C, D)	BCSC[5]
ER/HER2 joint distribution	Subtype distribution of ER/HER2 by age and stage or tumor size at diagnosis	BCSC[5], Mandelblatt et al. (2018)[1]
Mammography performance	Test sensitivity of initial and subsequent mammography by age (40-44, 45-49, 50-64, 65-74, 75-89), screening interval and mode of detection.	BCSC[5], Kerlikowske et al. (2022)[9]
Treatment dissemination	Treatments and rates of use by time period, ER/HER2 status, stage and age for initial breast cancer diagnosis	Mandelblatt et al. (2018)[1], Plevritis et al. (2018)[7], Caswell-Jin et al. (2024)[8]
Treatment effects	Clinical trials and meta-analyses of clinical trial results by ER/HER2 for efficacy of systemic primary and metastatic therapy, and of newer targeted therapies.	Caswell-Jin et al. (2024)[8], ECBCTG meta-analyses[10-15]

* Abbreviations: SEER: Surveillance, Epidemiology, and End Results; BI-RADS: Breast Imaging Reporting and Data System; BCSC: Breast Cancer Surveillance Consortium; ER: estrogen receptor; HER2: human epidermal growth factor receptor 2; AJCC: American Joint Committee on Cancer; ECBCTG: Early Breast Cancer Trialists' Collaborative Group

© 2026 Alagoz O et al. *JAMA Network Open.*

eTable 2. Summary of Key Model Features (Adopted from previous publications[1, 16, 17]

Feature	model E (Erasmus)	model W (Wisconsin)
Natural history of breast cancer	Tumor growth leading to fatal metastasis	Continuous tumor growth with some indolent and aggressive cases
Method of construction	Longitudinal, likelihood optimization, stochastic process, time to event	Longitudinal, stochastic process, state transition
Includes DCIS	Yes	Yes
Includes ER, HER2	Yes	Yes
Breast density representation	BI-RADS density (a,b,c,d) impacts risk of cancer and mammography performance	BI-RADS density (a,b,c,d) impacts risk of cancer and mammography performance
SEER breast cancer data used for model calibration	Incidence	Incidence and mortality
Screening benefit mechanism	Smaller tumor size, age shift	Smaller tumor size, age shift
Treatment benefit mechanism	Higher cure fraction	Higher cure fraction
Factors affecting treatment benefit	ER and HER2; age; year of and stage at diagnosis	ER and HER2; age; year of and stage at diagnosis

* Abbreviations: BI-RADS: DCIS: Ductal carcinoma in situ; ER: estrogen receptor; HER2: human epidermal growth factor receptor 2; SEER: Surveillance, Epidemiology, and End Results

Briefly, model E (Erasmus University) is a stochastic model utilizing a continuous tumor growth to simulate the natural history of breast cancer. The impact of screening relies on the size at detection, which assumes tumors diagnosed between the threshold size for screening and "fatal diameter" are cured, while those diagnosed at a size larger than the fatal tumor diameter lead to breast cancer death. The model includes four main components: demography, natural history of breast cancer, screening, and treatment.

Model W (University of Wisconsin-Madison) is a population-based discrete-event micro-simulation model coded in C++ [18]. Model W includes four main components: breast cancer natural history, detection, treatment and mortality. The model uses a cycle time of six months to represent the events experienced by each woman who enter the model at age 20. Model W assumes that a proportion of the tumors are of "limited malignant potential," and therefore does not lead to death from breast cancer.

© 2026 Alagoz O et al. *JAMA Network Open.*

eAppendix 2. List of Risk-Based Screening Strategies

eTable 3. List of Type B Risk-Based Screening Strategies

Strategy	Low-risk (≤ 0.8%)	Average-risk (0.81-1.79%)	Intermediate-risk (1.80-2.41%)	High-risk (≥ 2.42%)
**B1**	No Screening	Biennial	Biennial	Biennial
**B2**	No Screening	Biennial	Annual	Annual
**B3**	Triennial	Triennial	Biennial	Annual
**B4**	Triennial	Biennial	Biennial	Annual
**B5**	Triennial	Biennial	Annual	Annual
**B6**	Triennial	Biennial	Biennial	Biennial
**B7**	Biennial	Biennial	Biennial	Annual
**B8**	Biennial	Biennial	Annual	Annual
**B9**	Biennial	Annual	Annual	Annual

© 2026 Alagoz O et al. *JAMA Network Open.*

eTable 4. List of Type C Risk-Based Screening Strategies

Age	Low-risk (%)	Average-risk (%)	Intermediate-risk (%)	High-risk (%)
C1				
40-44	≤0.44 No Screening	0.45-0.82 No Screening	0.83-1.04 No Screening	≥1.05 Biennial
45-49	≤0.64 No Screening	0.65-1.18 No Screening	1.19-1.46 Biennial	≥1.47 Biennial
50-54	≤0.83 No Screening	0.84-1.47 Biennial	1.48-1.79 Annual	≥1.80 Annual
55-59	≤0.98 Biennial	0.99-1.69 Biennial	1.70-2.16 Annual	≥2.17 Annual
60-64	≤1.22 Biennial	1.23-2.03 Annual	2.04-2.65 Annual	≥2.66 Annual
65-69	≤1.39 Biennial	1.40-2.32 Annual	2.33-2.99 Annual	≥3.00 Annual
70-74	≤1.53 Biennial	1.54-2.47 Annual	2.48-3.26 Annual	≥3.27 Annual
C2				
40-44	≤0.44 No Screening	0.45-0.82 No Screening	0.83-1.04 No Screening	≥1.05 Biennial
45-49	≤0.64 No Screening	0.65-1.18 No Screening	1.19-1.46 Biennial	≥1.47 Biennial
50-54	≤0.83 No Screening	0.84-1.47 Biennial	1.48-1.79 Biennial	≥1.80 Biennial
55-59	≤0.98 Biennial	0.99-1.69 Biennial	1.70-2.16 Annual	≥2.17 Annual
60-64	≤1.22 Biennial	1.23-2.03 Annual	2.04-2.65 Annual	≥2.66 Annual
65-69	≤1.39 Biennial	1.40-2.32 Annual	2.33-2.99 Annual	≥3.00 Annual
70-74	≤1.53 Biennial	1.54-2.47 Annual	2.48-3.26 Annual	≥3.27 Annual
C3				
40-44	≤0.44 No Screening	0.45-0.82 No Screening	0.83-1.04 Biennial	≥1.05 Biennial
45-49	≤0.64 No Screening	0.65-1.18 No Screening	1.19-1.46 Biennial	≥1.47 Biennial
50-54	≤0.83 No Screening	0.84-1.47 Biennial	1.48-1.79 Biennial	≥1.80 Biennial
55-59	≤0.98 Biennial	0.99-1.69 Biennial	1.70-2.16 Biennial	≥2.17 Annual

© 2026 Alagoz O et al. *JAMA Network Open.*

60-64	≤1.22<br>Biennial	1.23-2.03<br>Annual	2.04-2.65<br>Annual	≥2.66<br>Annual
65-69	≤1.39<br>Biennial	1.40-2.32<br>Annual	2.33-2.99<br>Annual	≥3.00<br>Annual
70-74	≤1.53<br>Biennial	1.54-2.47<br>Annual	2.48-3.26<br>Annual	≥3.27<br>Annual
C4				
40-44	≤0.44<br>No Screening	0.45-0.82<br>No Screening	0.83-1.04<br>No Screening	≥1.05<br>Biennial
45-49	≤0.64<br>No Screening	0.65-1.18<br>No Screening	1.19-1.46<br>Biennial	≥1.47<br>Biennial
50-54	≤0.83<br>No Screening	0.84-1.47<br>Biennial	1.48-1.79<br>Biennial	≥1.80<br>Annual
55-59	≤0.98<br>Biennial	0.99-1.69<br>Biennial	1.70-2.16<br>Annual	≥2.17<br>Annual
60-64	≤1.22<br>Biennial	1.23-2.03<br>Annual	2.04-2.65<br>Annual	≥2.66<br>Annual
65-69	≤1.39<br>Biennial	1.40-2.32<br>Annual	2.33-2.99<br>Annual	≥3.00<br>Annual
70-74	≤1.53<br>Biennial	1.54-2.47<br>Annual	2.48-3.26<br>Annual	≥3.27<br>Annual
C5				
40-44	≤0.44<br>No Screening	0.45-0.82<br>No Screening	0.83-1.04<br>No Screening	≥1.05<br>Biennial
45-49	≤0.64<br>No Screening	0.65-1.18<br>No Screening	1.19-1.46<br>Biennial	≥1.47<br>Biennial
50-54	≤0.83<br>No Screening	0.84-1.47<br>Biennial	1.48-1.79<br>Biennial	≥1.80<br>Annual
55-59	≤0.98<br>Biennial	0.99-1.69<br>Biennial	1.70-2.16<br>Biennial	≥2.17<br>Annual
60-64	≤1.22<br>Biennial	1.23-2.03<br>Biennial	2.04-2.65<br>Biennial	≥2.66<br>Annual
65-69	≤1.39<br>Biennial	1.40-2.32<br>Biennial	2.33-2.99<br>Biennial	≥3.00<br>Annual
70-74	≤1.53<br>Biennial	1.54-2.47<br>Biennial	2.48-3.26<br>Biennial	≥3.27<br>Annual
C6				
40-44	≤0.44<br>No Screening	0.45-0.82<br>No Screening	0.83-1.04<br>No Screening	≥1.05<br>Biennial
45-49	≤0.64<br>No Screening	0.65-1.18<br>No Screening	1.19-1.46<br>Biennial	≥1.47<br>Biennial

© 2026 Alagoz O et al. *JAMA Network Open.*

50-54	≤0.83<br>No Screening	0.84-1.47<br>Biennial	1.48-1.79<br>Biennial	≥1.80<br>Biennial
55-59	≤0.98<br>Biennial	0.99-1.69<br>Biennial	1.70-2.16<br>Biennial	≥2.17<br>Annual
60-64	≤1.22<br>Biennial	1.23-2.03<br>Biennial	2.04-2.65<br>Biennial	≥2.66<br>Annual
65-69	≤1.39<br>Biennial	1.40-2.32<br>Biennial	2.33-2.99<br>Biennial	≥3.00<br>Annual
70-74	≤1.53<br>Biennial	1.54-2.47<br>Biennial	2.48-3.26<br>Biennial	≥3.27<br>Annual
C7				
40-44	≤0.44<br>No Screening	0.45-0.82<br>No Screening	0.83-1.04<br>No Screening	≥1.05<br>Biennial
45-49	≤0.64<br>No Screening	0.65-1.18<br>No Screening	1.19-1.46<br>Biennial	≥1.47<br>Biennial
50-54	≤0.83<br>No Screening	0.84-1.47<br>Biennial	1.48-1.79<br>Biennial	≥1.80<br>Biennial
55-59	≤0.98<br>Biennial	0.99-1.69<br>Biennial	1.70-2.16<br>Biennial	≥2.17<br>Annual
60-64	≤1.22<br>Biennial	1.23-2.03<br>Biennial	2.04-2.65<br>Biennial	≥2.66<br>Annual
65-69	≤1.39<br>Biennial	1.40-2.32<br>Biennial	2.33-2.99<br>Biennial	≥3.00<br>Annual
70-74	≤1.53<br>Biennial	1.54-2.47<br>Biennial	2.48-3.26<br>Annual	≥3.27<br>Annual
C8				
40-44	≤0.44<br>No Screening	0.45-0.82<br>No Screening	0.83-1.04<br>No Screening	≥1.05<br>Biennial
45-49	≤0.64<br>No Screening	0.65-1.18<br>No Screening	1.19-1.46<br>Biennial	≥1.47<br>Biennial
50-54	≤0.83<br>No Screening	0.84-1.47<br>Biennial	1.48-1.79<br>Biennial	≥1.80<br>Annual
55-59	≤0.98<br>Biennial	0.99-1.69<br>Biennial	1.70-2.16<br>Biennial	≥2.17<br>Annual
60-64	≤1.22<br>Biennial	1.23-2.03<br>Biennial	2.04-2.65<br>Biennial	≥2.66<br>Annual
65-69	≤1.39<br>Biennial	1.40-2.32<br>Biennial	2.33-2.99<br>Biennial	≥3.00<br>Annual
70-74	≤1.53<br>Biennial	1.54-2.47<br>Biennial	2.48-3.26<br>Annual	≥3.27<br>Annual
C9				

© 2026 Alagoz O et al. *JAMA Network Open.*

40-44	≤0.44 Triennial	0.45-0.82 Triennial	0.83-1.04 Triennial	≥1.05 Biennial
45-49	≤0.64 Triennial	0.65-1.18 Triennial	1.19-1.46 Biennial	≥1.47 Biennial
50-54	≤0.83 Triennial	0.84-1.47 Biennial	1.48-1.79 Annual	≥1.80 Annual
55-59	≤0.98 Biennial	0.99-1.69 Biennial	1.70-2.16 Annual	≥2.17 Annual
60-64	≤1.22 Biennial	1.23-2.03 Annual	2.04-2.65 Annual	≥2.66 Annual
65-69	≤1.39 Biennial	1.40-2.32 Annual	2.33-2.99 Annual	≥3.00 Annual
70-74	≤1.53 Biennial	1.54-2.47 Annual	2.48-3.26 Annual	≥3.27 Annual
C10				
40-44	≤0.44 Triennial	0.45-0.82 Triennial	0.83-1.04 Triennial	≥1.05 Biennial
45-49	≤0.64 Triennial	0.65-1.18 Triennial	1.19-1.46 Biennial	≥1.47 Biennial
50-54	≤0.83 Triennial	0.84-1.47 Biennial	1.48-1.79 Biennial	≥1.80 Biennial
55-59	≤0.98 Biennial	0.99-1.69 Biennial	1.70-2.16 Annual	≥2.17 Annual
60-64	≤1.22 Biennial	1.23-2.03 Annual	2.04-2.65 Annual	≥2.66 Annual
65-69	≤1.39 Biennial	1.40-2.32 Annual	2.33-2.99 Annual	≥3.00 Annual
70-74	≤1.53 Biennial	1.54-2.47 Annual	2.48-3.26 Annual	≥3.27 Annual
C11				
40-44	≤0.44 Triennial	0.45-0.82 Triennial	0.83-1.04 Triennial	≥1.05 Biennial
45-49	≤0.64 Triennial	0.65-1.18 Triennial	1.19-1.46 Biennial	≥1.47 Biennial
50-54	≤0.83 Triennial	0.84-1.47 Biennial	1.48-1.79 Biennial	≥1.80 Annual
55-59	≤0.98 Biennial	0.99-1.69 Biennial	1.70-2.16 Annual	≥2.17 Annual
60-64	≤1.22 Biennial	1.23-2.03 Annual	2.04-2.65 Annual	≥2.66 Annual
65-69	≤1.39 Biennial	1.40-2.32 Annual	2.33-2.99 Annual	≥3.00 Annual

© 2026 Alagoz O et al. *JAMA Network Open.*

70-74	≤1.53<br>Biennial	1.54-2.47<br>Annual	2.48-3.26<br>Annual	≥3.27<br>Annual
C12				
40-44	≤0.44<br>Biennial	0.45-0.82<br>Biennial	0.83-1.04<br>Biennial	≥1.05<br>Biennial
45-49	≤0.64<br>Biennial	0.65-1.18<br>Biennial	1.19-1.46<br>Biennial	≥1.47<br>Biennial
50-54	≤0.83<br>Biennial	0.84-1.47<br>Biennial	1.48-1.79<br>Annual	≥1.80<br>Annual
55-59	≤0.98<br>Biennial	0.99-1.69<br>Biennial	1.70-2.16<br>Annual	≥2.17<br>Annual
60-64	≤1.22<br>Biennial	1.23-2.03<br>Annual	2.04-2.65<br>Annual	≥2.66<br>Annual
65-69	≤1.39<br>Biennial	1.40-2.32<br>Annual	2.33-2.99<br>Annual	≥3.00<br>Annual
70-74	≤1.53<br>Biennial	1.54-2.47<br>Annual	2.48-3.26<br>Annual	≥3.27<br>Annual
C13				
40-44	≤0.44<br>Biennial	0.45-0.82<br>Biennial	0.83-1.04<br>Annual	≥1.05<br>Annual
45-49	≤0.64<br>Biennial	0.65-1.18<br>Biennial	1.19-1.46<br>Annual	≥1.47<br>Annual
50-54	≤0.83<br>Biennial	0.84-1.47<br>Biennial	1.48-1.79<br>Annual	≥1.80<br>Annual
55-59	≤0.98<br>Biennial	0.99-1.69<br>Biennial	1.70-2.16<br>Annual	≥2.17<br>Annual
60-64	≤1.22<br>Biennial	1.23-2.03<br>Annual	2.04-2.65<br>Annual	≥2.66<br>Annual
65-69	≤1.39<br>Biennial	1.40-2.32<br>Annual	2.33-2.99<br>Annual	≥3.00<br>Annual
70-74	≤1.53<br>Biennial	1.54-2.47<br>Annual	2.48-3.26<br>Annual	≥3.27<br>Annual
C14				
40-44	≤0.44<br>Biennial	0.45-0.82<br>Biennial	0.83-1.04<br>Biennial	≥1.05<br>Biennial
45-49	≤0.64<br>Biennial	0.65-1.18<br>Biennial	1.19-1.46<br>Biennial	≥1.47<br>Biennial
50-54	≤0.83<br>Biennial	0.84-1.47<br>Biennial	1.48-1.79<br>Annual	≥1.80<br>Annual
55-59	≤0.98<br>Biennial	0.99-1.69<br>Biennial	1.70-2.16<br>Annual	≥2.17<br>Annual

© 2026 Alagoz O et al. *JAMA Network Open.*

60-64	≤1.22<br>Annual	1.23-2.03<br>Annual	2.04-2.65<br>Annual	≥2.66<br>Annual
65-69	≤1.39<br>Annual	1.40-2.32<br>Annual	2.33-2.99<br>Annual	≥3.00<br>Annual
70-74	≤1.53<br>Annual	1.54-2.47<br>Annual	2.48-3.26<br>Annual	≥3.27<br>Annual
C15				
40-44	≤0.44<br>Biennial	0.45-0.82<br>Biennial	0.83-1.04<br>Annual	≥1.05<br>Annual
45-49	≤0.64<br>Biennial	0.65-1.18<br>Biennial	1.19-1.46<br>Annual	≥1.47<br>Annual
50-54	≤0.83<br>Biennial	0.84-1.47<br>Biennial	1.48-1.79<br>Annual	≥1.80<br>Annual
55-59	≤0.98<br>Biennial	0.99-1.69<br>Biennial	1.70-2.16<br>Annual	≥2.17<br>Annual
60-64	≤1.22<br>Annual	1.23-2.03<br>Annual	2.04-2.65<br>Annual	≥2.66<br>Annual
65-69	≤1.39<br>Annual	1.40-2.32<br>Annual	2.33-2.99<br>Annual	≥3.00<br>Annual
70-74	≤1.53<br>Annual	1.54-2.47<br>Annual	2.48-3.26<br>Annual	≥3.27<br>Annual
C16				
40-44	≤0.44<br>Biennial	0.45-0.82<br>Biennial	0.83-1.04<br>Biennial	≥1.05<br>Biennial
45-49	≤0.64<br>Biennial	0.65-1.18<br>Biennial	1.19-1.46<br>Annual	≥1.47<br>Annual
50-54	≤0.83<br>Biennial	0.84-1.47<br>Biennial	1.48-1.79<br>Annual	≥1.80<br>Annual
55-59	≤0.98<br>Biennial	0.99-1.69<br>Biennial	1.70-2.16<br>Annual	≥2.17<br>Annual
60-64	≤1.22<br>Biennial	1.23-2.03<br>Annual	2.04-2.65<br>Annual	≥2.66<br>Annual
65-69	≤1.39<br>Biennial	1.40-2.32<br>Annual	2.33-2.99<br>Annual	≥3.00<br>Annual
70-74	≤1.53<br>Biennial	1.54-2.47<br>Annual	2.48-3.26<br>Annual	≥3.27<br>Annual
C17				
40-44	≤0.44<br>No Screening	0.45-0.82<br>Biennial	0.83-1.04<br>Biennial	≥1.05<br>Annual
45-49	≤0.64<br>No Screening	0.65-1.18<br>Biennial	1.19-1.46<br>Biennial	≥1.47<br>Annual

© 2026 Alagoz O et al. *JAMA Network Open.*

50-54	≤0.83<br>No Screening	0.84-1.47<br>Biennial	1.48-1.79<br>Biennial	≥1.80<br>Annual
55-59	≤0.98<br>No Screening	0.99-1.69<br>Biennial	1.70-2.16<br>Biennial	≥2.17<br>Annual
60-64	≤1.22<br>No Screening	1.23-2.03<br>Biennial	2.04-2.65<br>Biennial	≥2.66<br>Annual
65-69	≤1.39<br>No Screening	1.40-2.32<br>Biennial	2.33-2.99<br>Biennial	≥3.00<br>Annual
70-74	≤1.53<br>No Screening	1.54-2.47<br>Biennial	2.48-3.26<br>Biennial	≥3.27<br>Annual
C18				
40-44	≤0.44<br>No Screening	0.45-0.82<br>No Screening	0.83-1.04<br>Biennial	≥1.05<br>Annual
45-49	≤0.64<br>No Screening	0.65-1.18<br>No Screening	1.19-1.46<br>Biennial	≥1.47<br>Annual
50-54	≤0.83<br>No Screening	0.84-1.47<br>No Screening	1.48-1.79<br>Biennial	≥1.80<br>Annual
55-59	≤0.98<br>No Screening	0.99-1.69<br>No Screening	1.70-2.16<br>Biennial	≥2.17<br>Annual
60-64	≤1.22<br>No Screening	1.23-2.03<br>No Screening	2.04-2.65<br>Biennial	≥2.66<br>Annual
65-69	≤1.39<br>No Screening	1.40-2.32<br>No Screening	2.33-2.99<br>Biennial	≥3.00<br>Annual
70-74	≤1.53<br>No Screening	1.54-2.47<br>No Screening	2.48-3.26<br>Biennial	≥3.27<br>Annual
C19				
40-44	≤0.44<br>No Screening	0.45-0.82<br>No Screening	0.83-1.04<br>Annual	≥1.05<br>Annual
45-49	≤0.64<br>No Screening	0.65-1.18<br>No Screening	1.19-1.46<br>Annual	≥1.47<br>Annual
50-54	≤0.83<br>No Screening	0.84-1.47<br>No Screening	1.48-1.79<br>Annual	≥1.80<br>Annual
55-59	≤0.98<br>No Screening	0.99-1.69<br>No Screening	1.70-2.16<br>Annual	≥2.17<br>Annual
60-64	≤1.22<br>No Screening	1.23-2.03<br>No Screening	2.04-2.65<br>Annual	≥2.66<br>Annual
65-69	≤1.39<br>No Screening	1.40-2.32<br>No Screening	2.33-2.99<br>Annual	≥3.00<br>Annual
70-74	≤1.53<br>No Screening	1.54-2.47<br>No Screening	2.48-3.26<br>Annual	≥3.27<br>Annual
C20				

© 2026 Alagoz O et al. *JAMA Network Open.*

40-44	≤0.44<br>No Screening	0.45-0.82<br>Triennial	0.83-1.04<br>Biennial	≥1.05<br>Annual
45-49	≤0.64<br>No Screening	0.65-1.18<br>Triennial	1.19-1.46<br>Biennial	≥1.47<br>Annual
50-54	≤0.83<br>No Screening	0.84-1.47<br>Triennial	1.48-1.79<br>Biennial	≥1.80<br>Annual
55-59	≤0.98<br>No Screening	0.99-1.69<br>Triennial	1.70-2.16<br>Biennial	≥2.17<br>Annual
60-64	≤1.22<br>No Screening	1.23-2.03<br>Triennial	2.04-2.65<br>Biennial	≥2.66<br>Annual
65-69	≤1.39<br>No Screening	1.40-2.32<br>Triennial	2.33-2.99<br>Biennial	≥3.00<br>Annual
70-74	≤1.53<br>No Screening	1.54-2.47<br>Triennial	2.48-3.26<br>Biennial	≥3.27<br>Annual
C21				
40-44	≤0.44<br>Triennial	0.45-0.82<br>Biennial	0.83-1.04<br>Annual	≥1.05<br>Annual
45-49	≤0.64<br>Triennial	0.65-1.18<br>Biennial	1.19-1.46<br>Annual	≥1.47<br>Annual
50-54	≤0.83<br>Triennial	0.84-1.47<br>Biennial	1.48-1.79<br>Annual	≥1.80<br>Annual
55-59	≤0.98<br>Triennial	0.99-1.69<br>Biennial	1.70-2.16<br>Annual	≥2.17<br>Annual
60-64	≤1.22<br>Triennial	1.23-2.03<br>Biennial	2.04-2.65<br>Annual	≥2.66<br>Annual
65-69	≤1.39<br>Triennial	1.40-2.32<br>Biennial	2.33-2.99<br>Annual	≥3.00<br>Annual
70-74	≤1.53<br>Triennial	1.54-2.47<br>Biennial	2.48-3.26<br>Annual	≥3.27<br>Annual
C22				
40-44	≤0.44<br>Triennial	0.45-0.82<br>Biennial	0.83-1.04<br>Biennial	≥1.05<br>Annual
45-49	≤0.64<br>Triennial	0.65-1.18<br>Biennial	1.19-1.46<br>Biennial	≥1.47<br>Annual
50-54	≤0.83<br>Triennial	0.84-1.47<br>Biennial	1.48-1.79<br>Biennial	≥1.80<br>Annual
55-59	≤0.98<br>Triennial	0.99-1.69<br>Biennial	1.70-2.16<br>Biennial	≥2.17<br>Annual
60-64	≤1.22<br>Triennial	1.23-2.03<br>Biennial	2.04-2.65<br>Biennial	≥2.66<br>Annual
65-69	≤1.39<br>Triennial	1.40-2.32<br>Biennial	2.33-2.99<br>Biennial	≥3.00<br>Annual

© 2026 Alagoz O et al. *JAMA Network Open.*

70-74	≤1.53 Triennial	1.54-2.47 Biennial	2.48-3.26 Biennial	≥3.27 Annual
C23				
40-44	≤0.44 Triennial	0.45-0.82 Triennial	0.83-1.04 Biennial	≥1.05 Annual
45-49	≤0.64 Triennial	0.65-1.18 Triennial	1.19-1.46 Biennial	≥1.47 Annual
50-54	≤0.83 Triennial	0.84-1.47 Triennial	1.48-1.79 Biennial	≥1.80 Annual
55-59	≤0.98 Triennial	0.99-1.69 Triennial	1.70-2.16 Biennial	≥2.17 Annual
60-64	≤1.22 Triennial	1.23-2.03 Triennial	2.04-2.65 Biennial	≥2.66 Annual
65-69	≤1.39 Triennial	1.40-2.32 Triennial	2.33-2.99 Biennial	≥3.00 Annual
70-74	≤1.53 Triennial	1.54-2.47 Triennial	2.48-3.26 Biennial	≥3.27 Annual
C24				
40-44	≤0.44 Triennial	0.45-0.82 Triennial	0.83-1.04 Annual	≥1.05 Annual
45-49	≤0.64 Triennial	0.65-1.18 Triennial	1.19-1.46 Annual	≥1.47 Annual
50-54	≤0.83 Triennial	0.84-1.47 Triennial	1.48-1.79 Annual	≥1.80 Annual
55-59	≤0.98 Triennial	0.99-1.69 Triennial	1.70-2.16 Annual	≥2.17 Annual
60-64	≤1.22 Triennial	1.23-2.03 Triennial	2.04-2.65 Annual	≥2.66 Annual
65-69	≤1.39 Triennial	1.40-2.32 Triennial	2.33-2.99 Annual	≥3.00 Annual
70-74	≤1.53 Triennial	1.54-2.47 Triennial	2.48-3.26 Annual	≥3.27 Annual

© 2026 Alagoz O et al. *JAMA Network Open.*

eTable 5. List of Type D Risk-Based Screening Strategies

Strategy	Low-risk (≤ 0.8%)	Average-risk (0.81-1.79%)	Intermediate-risk (1.80-2.41%)	High-risk (≥ 2.42%)
**D1**	No Screening	Biennial	Biennial	Biennial
**D2**	No Screening	Biennial	Annual	Annual
**D3**	Triennial	Triennial	Biennial	Annual
**D4**	Triennial	Biennial	Biennial	Annual
**D5**	Triennial	Biennial	Annual	Annual
**D6**	Triennial	Biennial	Biennial	Biennial
**D7**	Biennial	Biennial	Biennial	Annual
**D8**	Biennial	Biennial	Annual	Annual
**D9**	Biennial	Annual	Annual	Annual

© 2026 Alagoz O et al. *JAMA Network Open.*

eTable 6. List of Type E Risk-Based Screening Strategies

Age	Low-risk (%)	Average-risk (%)	Intermediate-risk (%)	High-risk (%)
**E1**				
50-54	≤0.83 Biennial	0.84-1.47 Biennial	1.48-1.79 Annual	≥1.80 Annual
55-59	≤0.98 Biennial	0.99-1.69 Biennial	1.70-2.16 Annual	≥2.17 Annual
60-64	≤1.22 Biennial	1.23-2.03 Annual	2.04-2.65 Annual	≥2.66 Annual
65-69	≤1.39 Biennial	1.40-2.32 Annual	2.33-2.99 Annual	≥3.00 Annual
70-74	≤1.53 Biennial	1.54-2.47 Annual	2.48-3.26 Annual	≥3.27 Annual
**E2**				
50-54	≤0.83 No Screening	0.84-1.47 No Screening	1.48-1.79 Biennial	≥1.80 Annual
55-59	≤0.98 No Screening	0.99-1.69 No Screening	1.70-2.16 Biennial	≥2.17 Annual
60-64	≤1.22 No Screening	1.23-2.03 No Screening	2.04-2.65 Biennial	≥2.66 Annual
65-69	≤1.39 No Screening	1.40-2.32 No Screening	2.33-2.99 Biennial	≥3.00 Annual
70-74	≤1.53 No Screening	1.54-2.47 No Screening	2.48-3.26 Biennial	≥3.27 Annual
E3				
50-54	≤0.83 Triennial	0.84-1.47 Biennial	1.48-1.79 Biennial	≥1.80 Biennial
55-59	≤0.98 Biennial	0.99-1.69 Biennial	1.70-2.16 Annual	≥2.17 Annual
60-64	≤1.22 Biennial	1.23-2.03 Annual	2.04-2.65 Annual	≥2.66 Annual
65-69	≤1.39 Biennial	1.40-2.32 Annual	2.33-2.99 Annual	≥3.00 Annual
70-74	≤1.53 Biennial	1.54-2.47 Annual	2.48-3.26 Annual	≥3.27 Annual
E4				
50-54	≤0.83 Biennial	0.84-1.47 Biennial	1.48-1.79 Annual	≥1.80 Annual

© 2026 Alagoz O et al. *JAMA Network Open.*

55-59	≤0.98<br>Biennial	0.99-1.69<br>Biennial	1.70-2.16<br>Annual	≥2.17<br>Annual
60-64	≤1.22<br>Annual	1.23-2.03<br>Annual	2.04-2.65<br>Annual	≥2.66<br>Annual
65-69	≤1.39<br>Annual	1.40-2.32<br>Annual	2.33-2.99<br>Annual	≥3.00<br>Annual
70-74	≤1.53<br>Annual	1.54-2.47<br>Annual	2.48-3.26<br>Annual	≥3.27<br>Annual
E5				
50-54	≤0.83<br>Triennial	0.84-1.47<br>Triennial	1.48-1.79<br>Biennial	≥1.80<br>Annual
55-59	≤0.98<br>Triennial	0.99-1.69<br>Triennial	1.70-2.16<br>Biennial	≥2.17<br>Annual
60-64	≤1.22<br>Triennial	1.23-2.03<br>Triennial	2.04-2.65<br>Biennial	≥2.66<br>Annual
65-69	≤1.39<br>Triennial	1.40-2.32<br>Triennial	2.33-2.99<br>Biennial	≥3.00<br>Annual
70-74	≤1.53<br>Triennial	1.54-2.47<br>Triennial	2.48-3.26<br>Biennial	≥3.27<br>Annual

© 2026 Alagoz O et al. *JAMA Network Open.*

eAppendix 3. Internal Validation of the Models

We conducted two internal validation analyses to demonstrate that the extended models accurately reproduce absolute risks. In the first analysis, we compared key breast cancer outcomes produced by the models -with and without the incorporation of absolute risk- under three screening strategies (no screening, biennial screening ages 50–74, and biennial screening ages 40–74). eTable 7 shows the results of this experiment, which show that the versions of the models that incorporated absolute risk projected nearly identical outcomes with those that did not incorporate the absolute risk.

© 2026 Alagoz O et al. *JAMA Network Open.*

eTable 7. Comparison of Key Breast Cancer Outcomes Produced by the CISNET Models With and Without the Incorporation of Absolute Risk Under Different Screening Strategies. All outcomes are expressed per 1000 screened women.

Model	Screening scenario	Model version	Number of mammo grams	Number of detected breast cancers	Number of breast cancer deaths	Expected life years	Life-years gained	Breast cancer deaths averted	Percent reduction in breast cancer mortality
Model E	No Screening	No absolute risk	0	116	26.0	42,725	0.0	0	0
	No Screening	Absolute risk	0	115	25.7	42,727	0.0	0	0
	B40-74	No absolute risk	16,084	129	17.6	42,878	153.4	8.4	32
	B40-74	Absolute risk	16,086	127	17.6	42,877	152.3	8.4	32
	B50-74	No absolute risk	11,192	126	18.9	42,840	115.0	7.1	27
	B50-74	Absolute risk	11,194	125	18.9	42,839	114.9	7.1	27
Model W	No Screening	No absolute risk	0	135	23.7	43,119	0.0	0	0
	No Screening	Absolute risk	0	135	23.6	43,121	0.0	0	0
	B40-74	No absolute risk	15,826	172	18.2	43,224	105.2	5.5	23
	B40-74	Absolute risk	15,835	171	18.2	43,225	106.8	5.5	23
	B50-74	No absolute risk	10,976	167	19.1	43,197	78.3	4.6	19
	B50-74	Absolute risk	10,983	167	19.1	43,198	79.5	4.6	19

In the second internal validation experiment, we compared the relative risk of breast cancer across risk deciles within each age and density group to the corresponding estimates from the BCSC risk calculator under different screening strategies. **eFigure 1** presents these comparisons for both models across the four density categories for ages 40-44 under no screening scenario; similar comparisons were conducted for all age groups and the other two screening strategies. As shown in **eFigure 1**, the differences between the model projections and the BCSC estimates were minimal, supporting the validity of the model extension.

© 2026 Alagoz O et al. *JAMA Network Open.*

eFigure 1. Comparison of Model-Projected Relative Risks and BCSC Risk Calculator Estimates Across Density Categories for Women Aged 40–44 Under no Screening Strategy. A) Density a; B) Density b; C) Density c; D) Density D.

A)

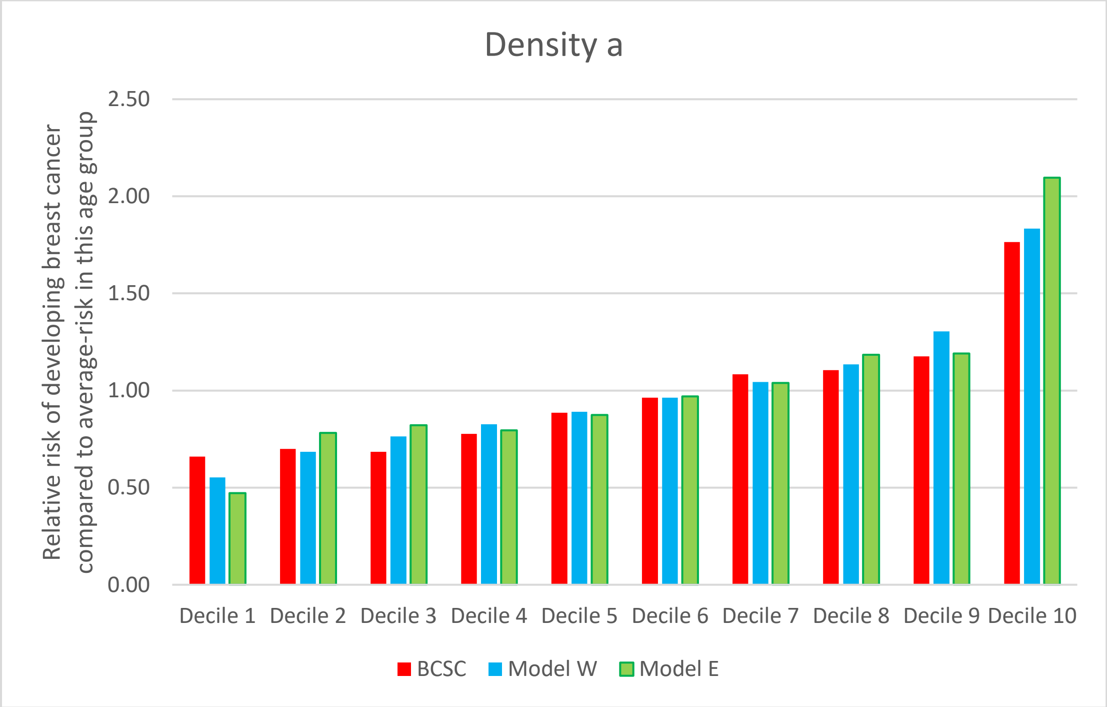

B)

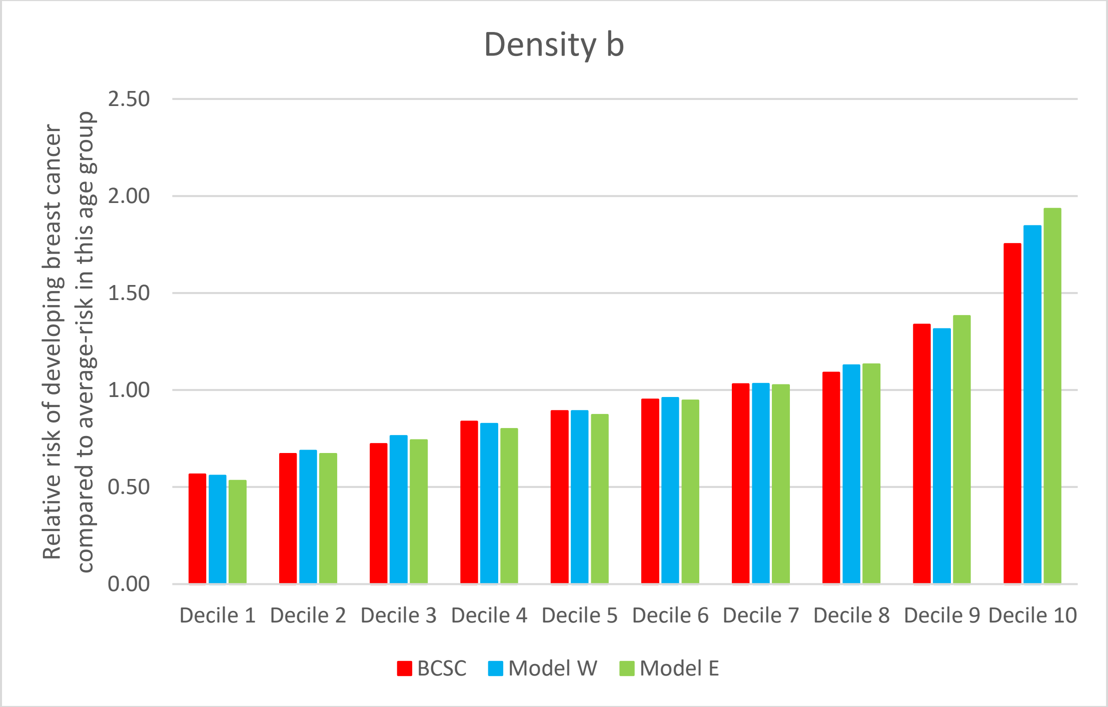

© 2026 Alagoz O et al. *JAMA Network Open.*

C)

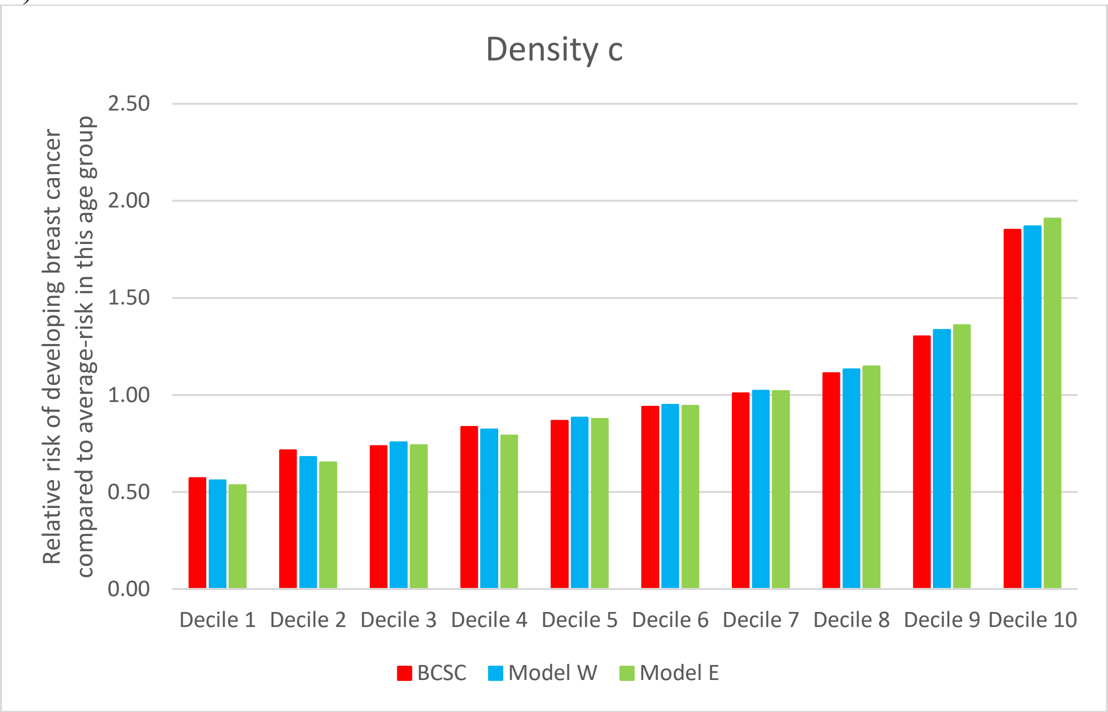

D)

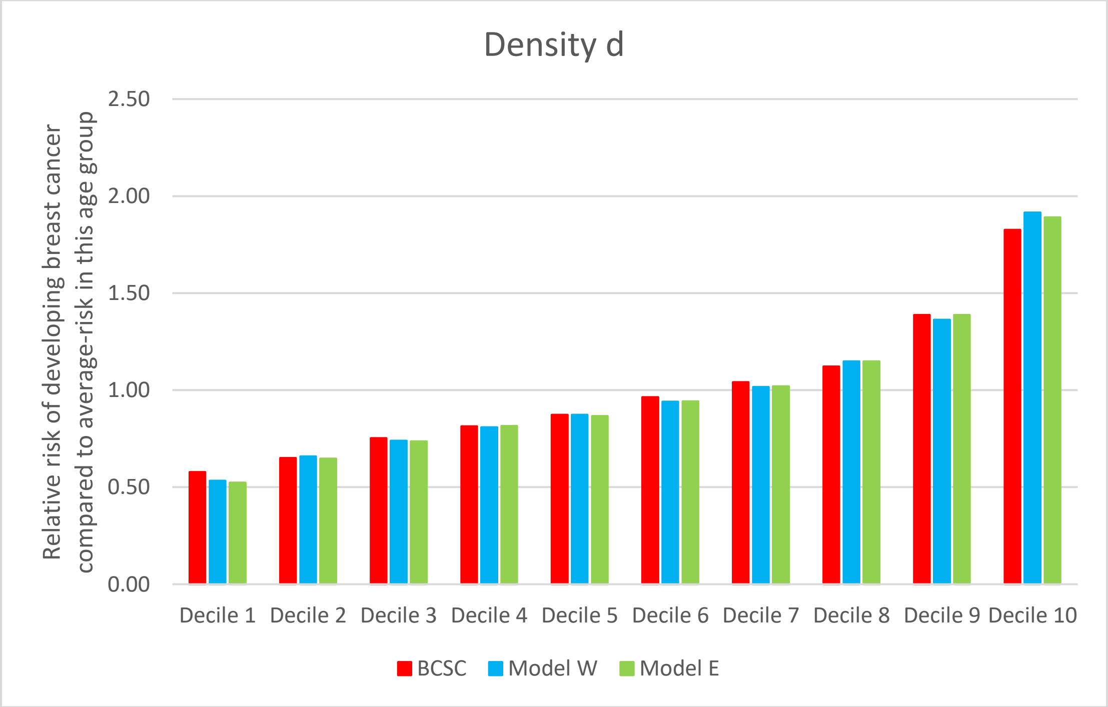

© 2026 Alagoz O et al. *JAMA Network Open.*

eAppendix 4. Additional Numerical Results

eTable 8. Estimated Outcomes Associated With Risk-Based Screening Strategies Compared to Biennial Screening for Ages 40-74 and 50-74 and Annual Screening for Ages 40-74 According to the Average Model Outcome. The values in parentheses represent outcomes predicted by model W and model E respectively.

Strategy	Number of mammograms	Number of FPs	Benign biopsies	Deaths averted	LYs gained
**Biennial 40-74**	15,960 (15835-16086)	1,365 (1355-1374)	200 (198-201)	6.8 (5.5-8.2)	127 (105-150)
**Biennial 50-74**	11,088 (10983-11194)	864 (856-872)	135 (133-136)	5.7 (4.5-6.9)	95 (77-112)
**Annual 40-74**	30,877 (30591-31162)	2,074 (2057-2091)	285 (283-288)	9.2 (7.7-10.6)	176 (151-201)
**B1**	12,868 (12761-12976)	1,105 (1097-1114)	167 (166-168)	6.2 (4.9-7.5)	112 (92-132)
**B2**	16,434 (16410-16457)	1,257 (1256-1258)	186 (185-186)	7.2 (6-8.4)	128 (109-148)
**B3**	13,353 (13333-13374)	1,302 (1301-1302)	210 (209-210)	6.2 (5-7.4)	112 (93-131)
**B4**	15,978 (15916-16041)	1,366 (1364-1367)	205 (205-206)	7 (5.7-8.3)	128 (107-150)
**B5**	18,153 (18195-18110)	1,460 (1467-1453)	217 (218-216)	7.5 (6.2-8.8)	137 (116-158)
**B6**	14,605 (14552-14658)	1,310 (1309-1312)	199 (198-199)	6.6 (5.2-7.9)	121 (100-143)
**B7**	17,318 (17168-17469)	1,419 (1409-1429)	207 (205-208)	7.2 (5.9-8.6)	134 (112-156)
**B8**	19,439 (19340-19537)	1,510 (1504-1516)	218 (217-219)	7.7 (6.4-9.1)	143 (120-165)
**B9**	27,225 (27132-27318)	1,880 (1877-1883)	263 (263-264)	8.9 (7.5-10.3)	168 (144-192)
**C1**	17,386 (17262-17510)	1,203 (1195-1211)	177 (176-178)	7.2 (6-8.4)	126 (106-145)
**C2**	16,911 (16857-16965)	1,180 (1177-1183)	174 (174-175)	7.1 (5.9-8.3)	123 (104-141)
**C3**	16,805 (16721-16888)	1,186 (1181-1192)	175 (174-176)	7.1 (5.9-8.3)	123 (105-141)
**C4**	17,119 (17023-17215)	1,190 (1184-1196)	176 (175-176)	7.2 (5.9-8.4)	124 (105-143)
**C5**	13,217 (13092-13342)	1,043 (1034-1052)	157 (156-159)	6.3 (5.1-7.6)	112 (92-131)
**C6**	13,009 (12927-13092)	1,033 (1026-1039)	156 (155-157)	6.3 (5.1-7.5)	110 (92-129)
**C7**	13,233 (13166-13300)	1,041 (1035-1046)	157 (156-158)	6.4 (5.2-7.5)	111 (93-129)
**C8**	13,441 (13331-13550)	1,051 (1042-1059)	158 (157-159)	6.4 (5.2-7.6)	112 (94-131)
**C9**	19,448 (19320-19577)	1,490 (1484-1496)	223 (222-224)	7.7 (6.3-9)	138 (116-159)
**C10**	18,966 (18942-18989)	1,467 (1468-1467)	220 (220-220)	7.5 (6.2-8.8)	135 (114-156)
**C11**	19,173 (19107-19240)	1,477 (1475-1480)	222 (221-222)	7.6 (6.3-8.9)	136 (115-158)

© 2026 Alagoz O et al. *JAMA Network Open.*

**C12**	21,194 (20992-21395)	1,568 (1556-1580)	225 (223-227)	8 (6.6-9.4)	147 (124-170)
**C13**	22,448 (22239-22657)	1,662 (1649-1675)	236 (235-238)	8.2 (6.9-9.6)	154 (131-177)
**C14**	22,561 (22344-22778)	1,605 (1593-1618)	229 (227-231)	8.2 (6.8-9.5)	149 (126-172)
**C15**	23,815 (23592-24039)	1,699 (1686-1713)	241 (239-243)	8.4 (7-9.8)	156 (133-179)
**C16**	21,812 (21731-21893)	1,611 (1610-1613)	230 (230-231)	8.1 (6.8-9.5)	150 (128-173)
**C17**	14,203 (14069-14336)	1,238 (1229-1247)	183 (181-184)	6.4 (5.2-7.6)	121 (101-141)
**C18**	6,742 (6682-6801)	640 (635-644)	100 (99-100)	3.4 (2.8-3.9)	66 (57-75)
**C19**	8,778 (8674-8882)	745 (738-752)	113 (112-114)	3.8 (3.2-4.4)	74 (64-84)
**C20**	11,664 (11569-11759)	1,171 (1164-1178)	187 (186-188)	5.6 (4.5-6.7)	107 (88-125)
**C21**	18,429 (18301-18557)	1,543 (1534-1551)	227 (225-228)	7.5 (6.1-8.8)	141 (119-164)
**C22**	16,410 (16310-16509)	1,438 (1430-1446)	214 (212-215)	7 (5.7-8.4)	133 (111-155)
**C23**	13,843 (13755-13932)	1,369 (1361-1377)	217 (216-219)	6.3 (5-7.5)	118 (97-139)
**C24**	15,861 (15746-15975)	1,474 (1465-1484)	230 (229-232)	6.7 (5.4-8)	127 (106-148)
**D1**	10,278 (10178-10378)	823 (815-831)	129 (128-130)	5.5 (4.4-6.7)	91 (75-108)
**D2**	13,566 (13475-13657)	954 (948-961)	145 (144-146)	6.4 (5.3-7.5)	105 (89-121)
**D3**	10,123 (10156-10090)	864 (864-864)	143 (142-143)	5.5 (4.4-6.5)	89 (74-104)
**D4**	12,055 (11935-12174)	908 (899-917)	141 (140-142)	6.1 (4.9-7.2)	100 (83-117)
**D5**	14,008 (13926-14091)	987 (981-993)	151 (150-152)	6.5 (5.4-7.7)	108 (91-124)
**D6**	10,721 (10630-10811)	855 (848-863)	134 (133-135)	5.6 (4.5-6.8)	94 (77-111)
**D7**	12,422 (12288-12557)	917 (908-927)	141 (140-143)	6.1 (5-7.3)	102 (84-119)
**D8**	14,372 (14271-14474)	996 (989-1003)	151 (150-152)	6.6 (5.4-7.7)	109 (92-126)
**D9**	20,224 (20051-20398)	1,232 (1221-1242)	180 (179-182)	7.5 (6.2-8.7)	126 (107-145)
**E1**	16,347 (16172-16522)	1,072 (1061-1083)	161 (159-162)	6.9 (5.7-8.1)	115 (97-133)
**E2**	4,766 (4715-4817)	442 (438-446)	75 (74-76)	2.9 (2.4-3.5)	52 (43-60)
**E3**	15,836 (15787-15886)	1,050 (1047-1053)	158 (158-159)	6.8 (5.6-8)	112 (96-129)
**E4**	17,718 (17529-17906)	1,109 (1098-1120)	165 (163-166)	7.1 (5.9-8.3)	117 (99-135)
**E5**	9,665 (9591-9740)	866 (859-873)	144 (143-145)	5.3 (4.2-6.4)	88 (72-104)

© 2026 Alagoz O et al. *JAMA Network Open.*

Notes. FP: false-positive; LY: life year.

© 2026 Alagoz O et al. *JAMA Network Open.*

eFigure 2. Efficiency Frontiers for the Estimated Lifetime Number of False-Positive Mammograms and Breast Cancer Deaths Averted for a Cohort of 1000 Women According to Model and Screening Strategy. A) Model W B) Model E

A)

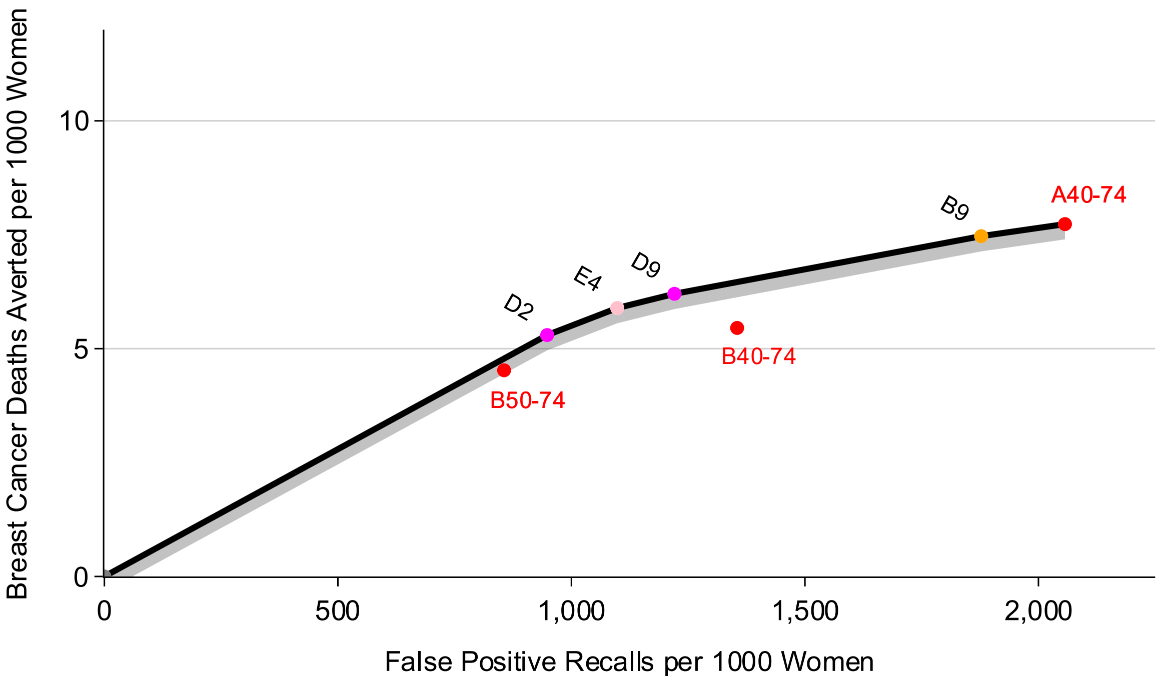

B)

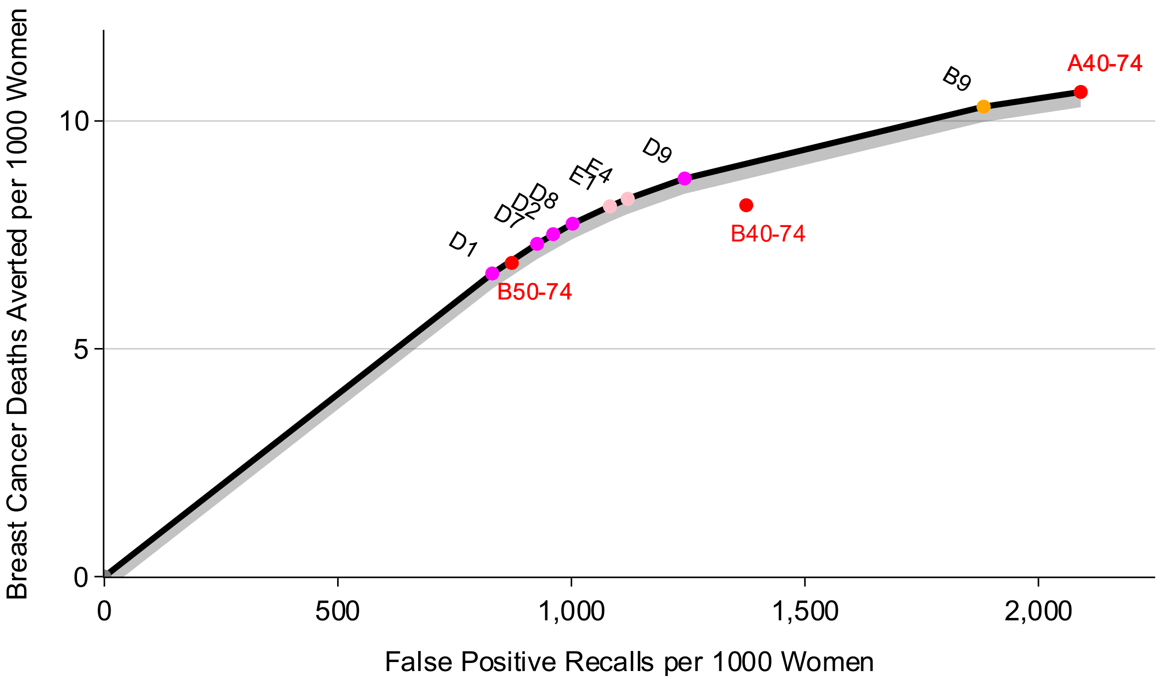

© 2026 Alagoz O et al. *JAMA Network Open.*

eFigure 3. Efficiency Frontiers for the Estimated Lifetime Number of False-Positive Mammograms and Life-Years Gained According to Model and Screening Strategy for a Cohort of 1000 Women. A) Model W B) Model E

A)

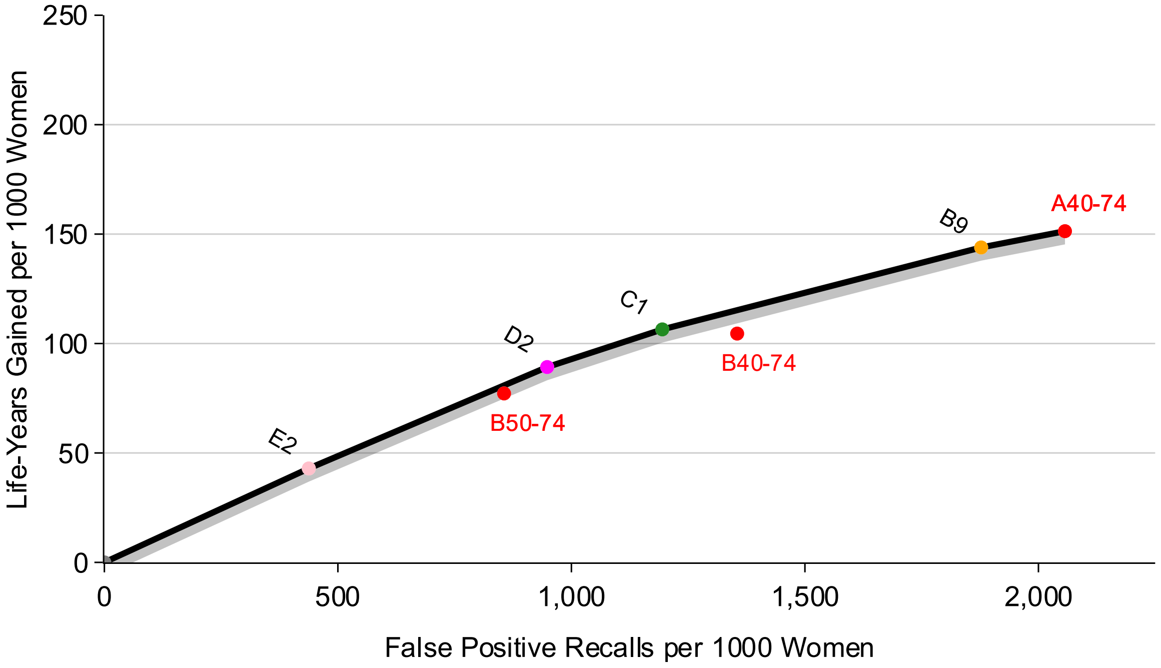

B)

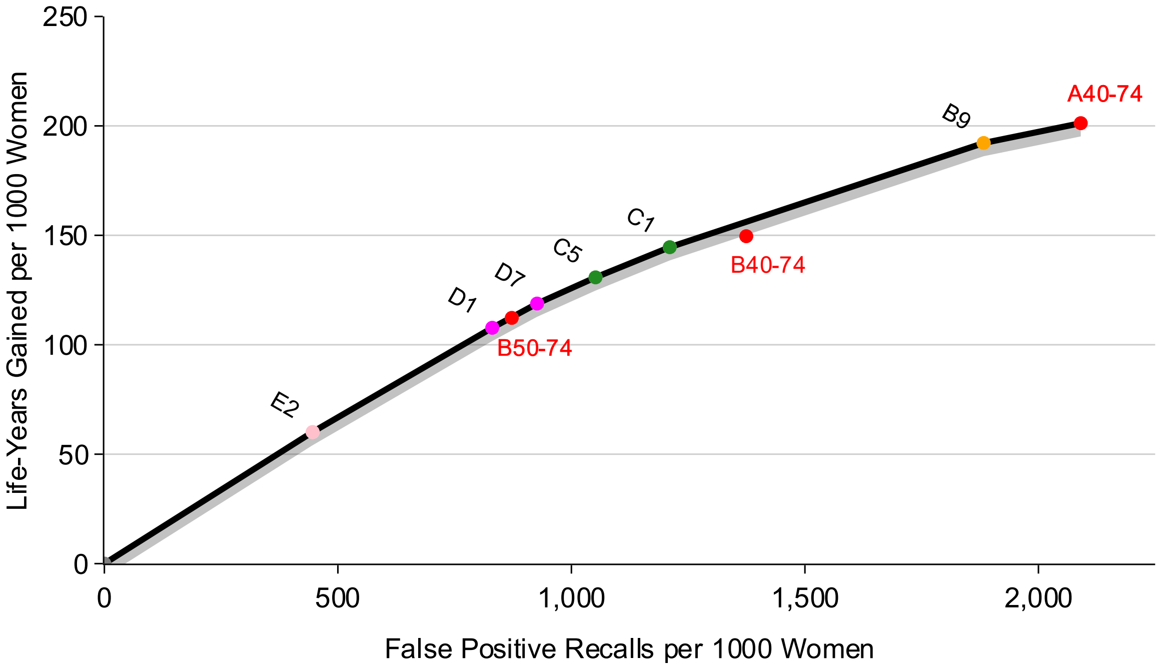

© 2026 Alagoz O et al. *JAMA Network Open.*

eFigure 4. Efficiency Frontiers for the Estimated Lifetime Number of Mammograms and Breast Cancer Deaths Averted for a Cohort of 1000 Women According to Model and Screening Strategy. A) Model W B) Model E

A)

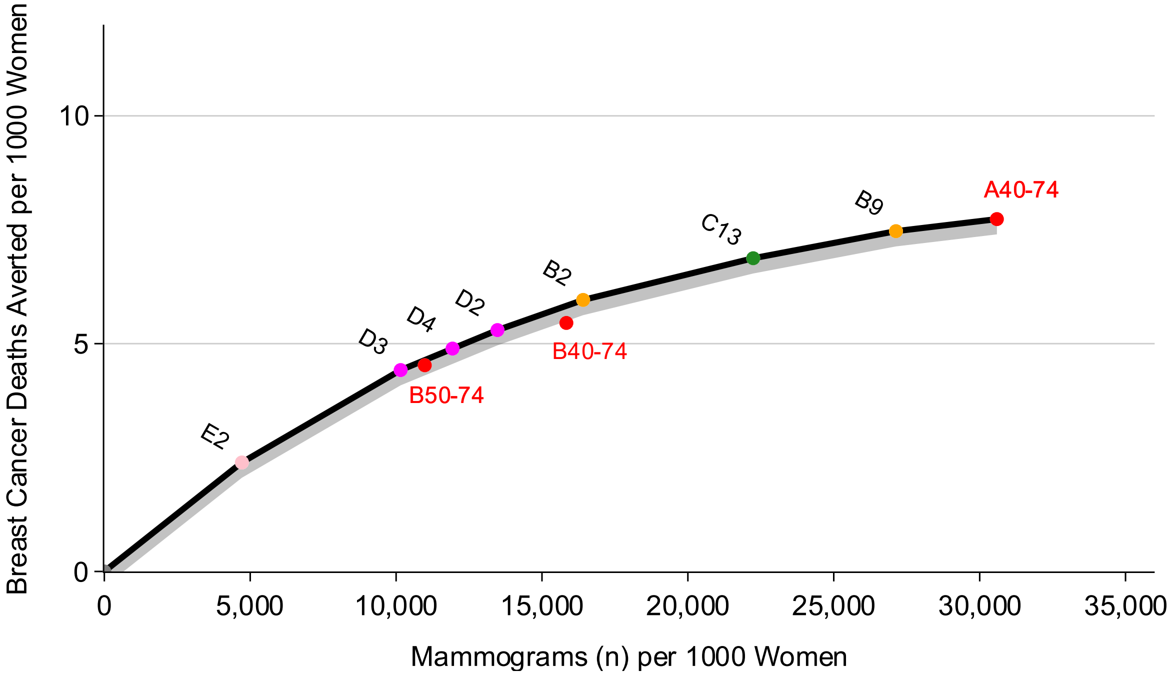

B)

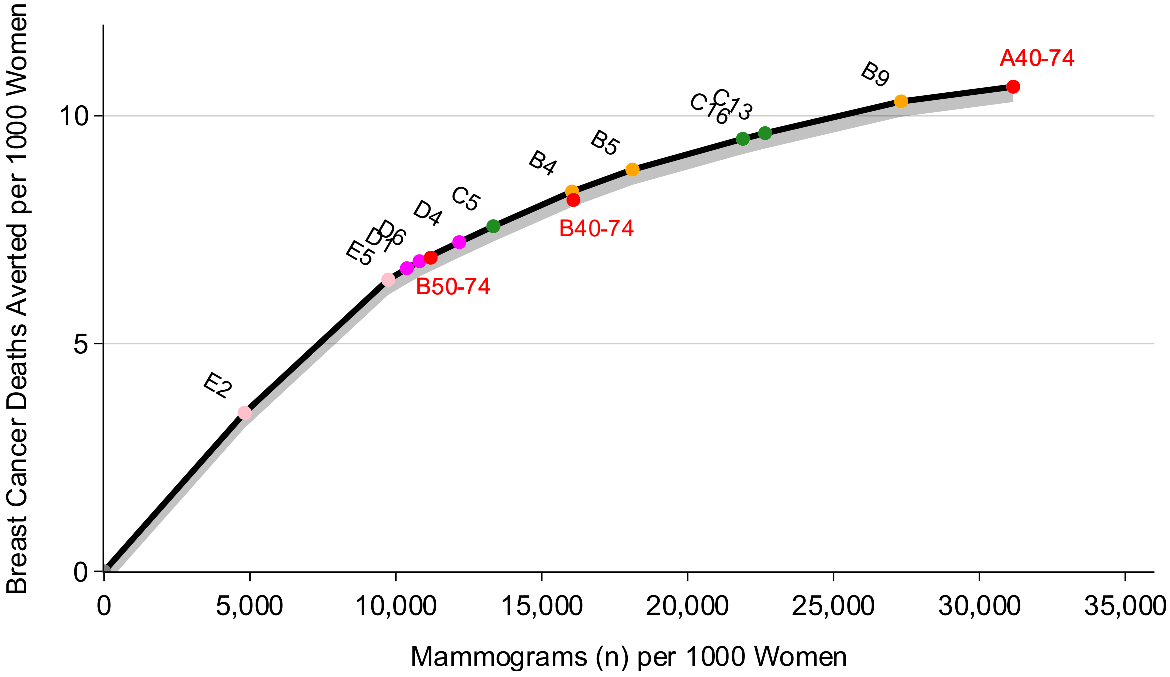

© 2026 Alagoz O et al. *JAMA Network Open.*

eFigure 5. Efficiency Frontiers for the Estimated Lifetime Number of Mammograms and Life-Years Gained According to Model and Screening Strategy for a Cohort of 1000 Women. A) Model W B) Model E

A)

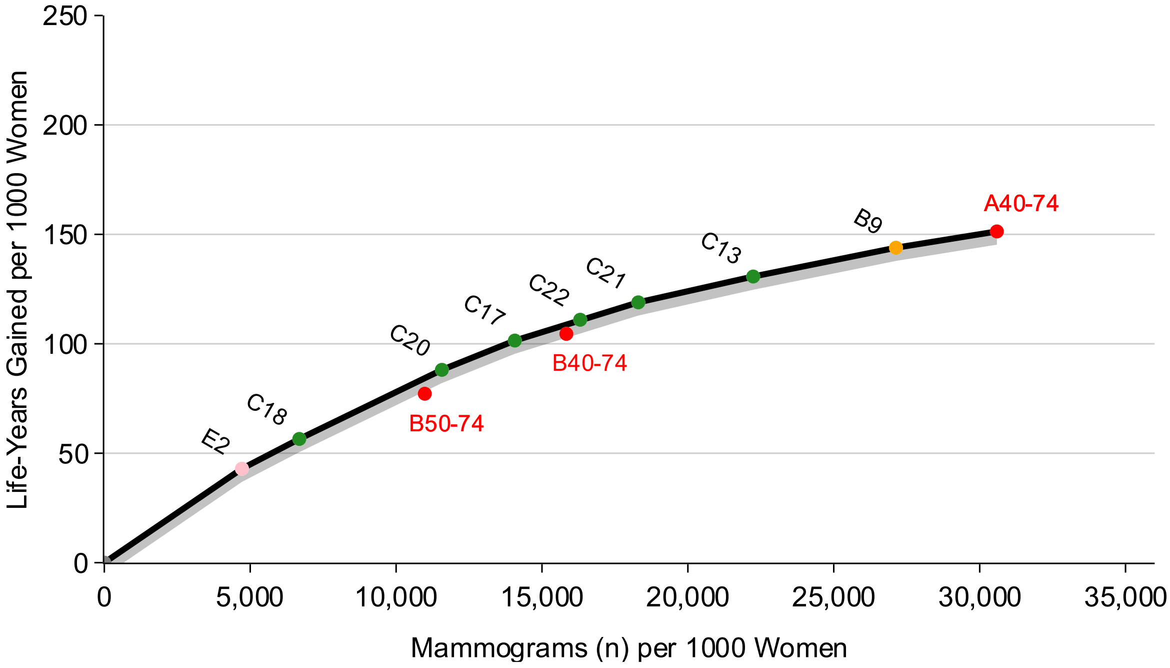

B)

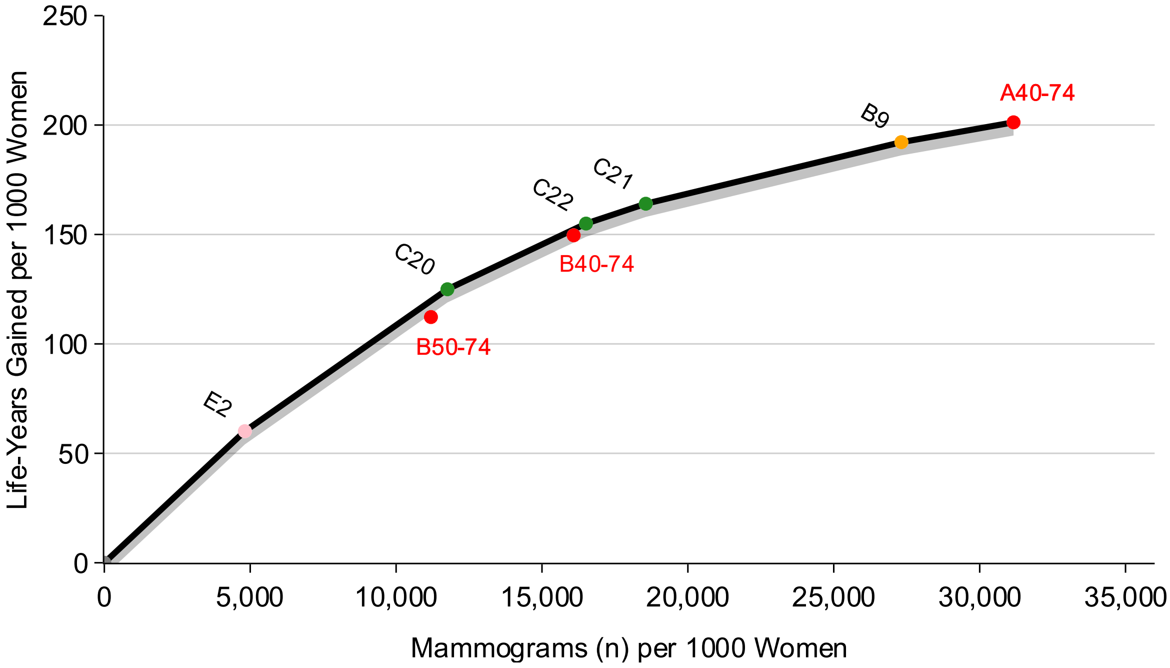

© 2026 Alagoz O et al. *JAMA Network Open.*

eFigure 6. Efficiency Frontiers for the Estimated Lifetime Number of False-Positive Mammograms and Percent Reduction in Breast Cancer Mortality for a Cohort of 1000 Women by Screening Strategy. The outcomes in these graphs are obtained by taking the average of the outcomes estimated by model W and model E.

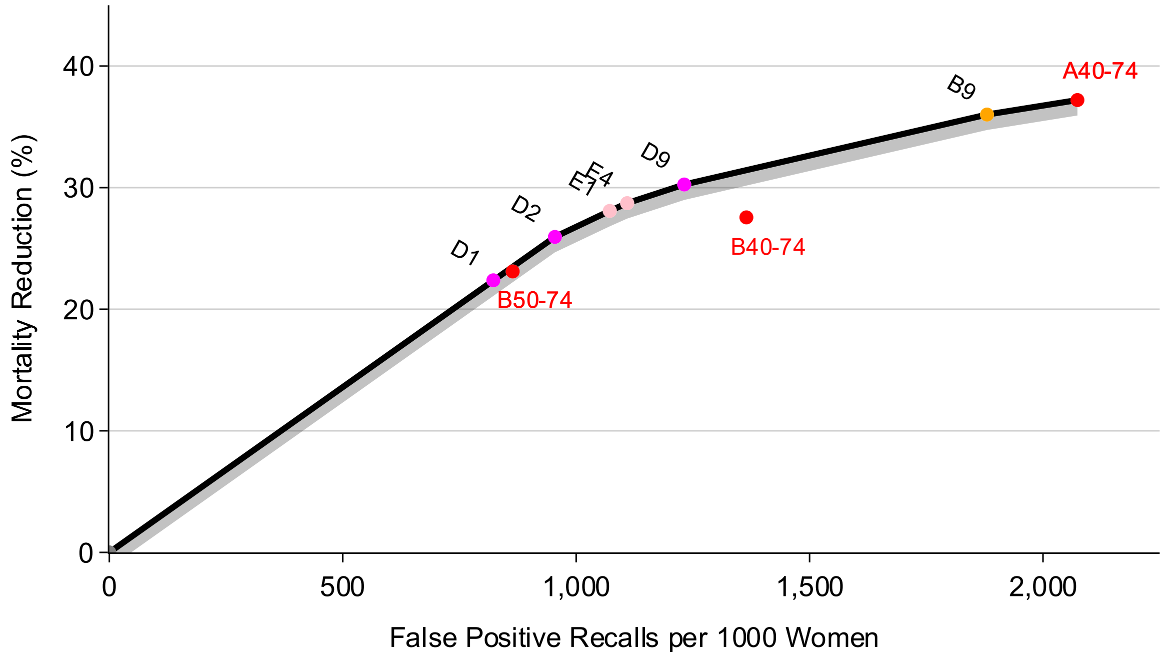

© 2026 Alagoz O et al. *JAMA Network Open.*

eFigure 7. Efficiency Frontiers for the Estimated Lifetime Number of Benign Biopsies, Life-Years Gained, and Breast Cancer Deaths Averted for a Cohort of 1000 Women by Screening Strategy. The outcomes in these graphs are obtained by taking the average of the outcomes estimated by model W and model E. A) Deaths averted vs. benign biopsies B) Life-years gained vs. benign biopsies

A)

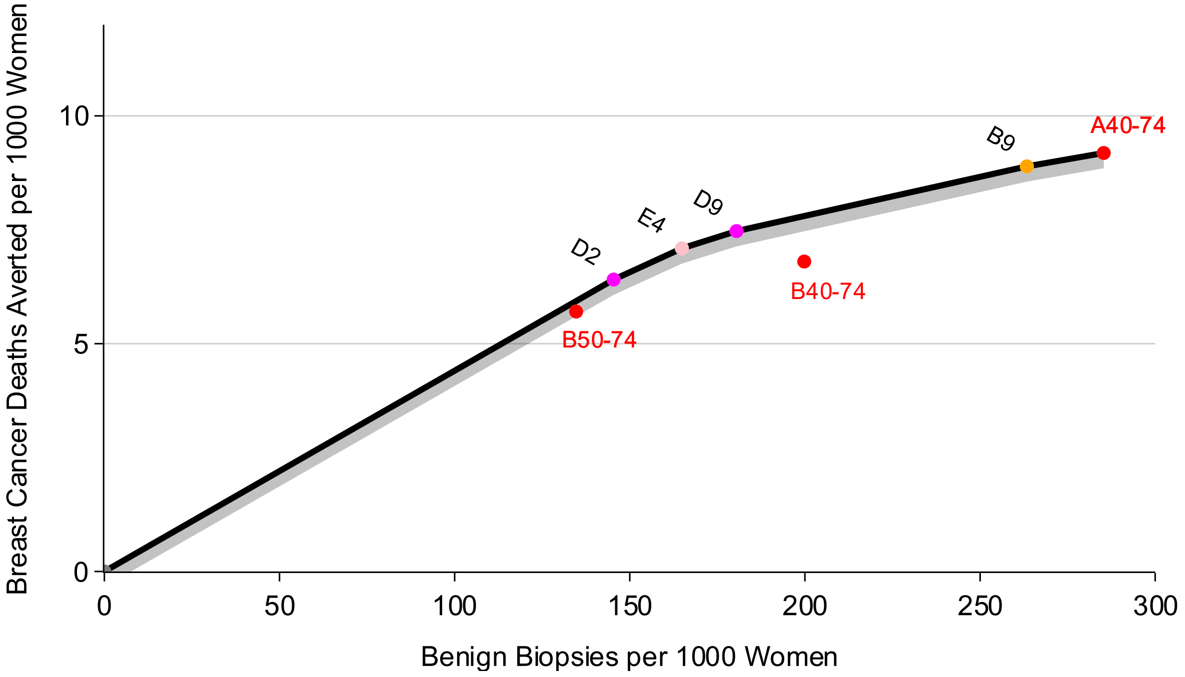

B)

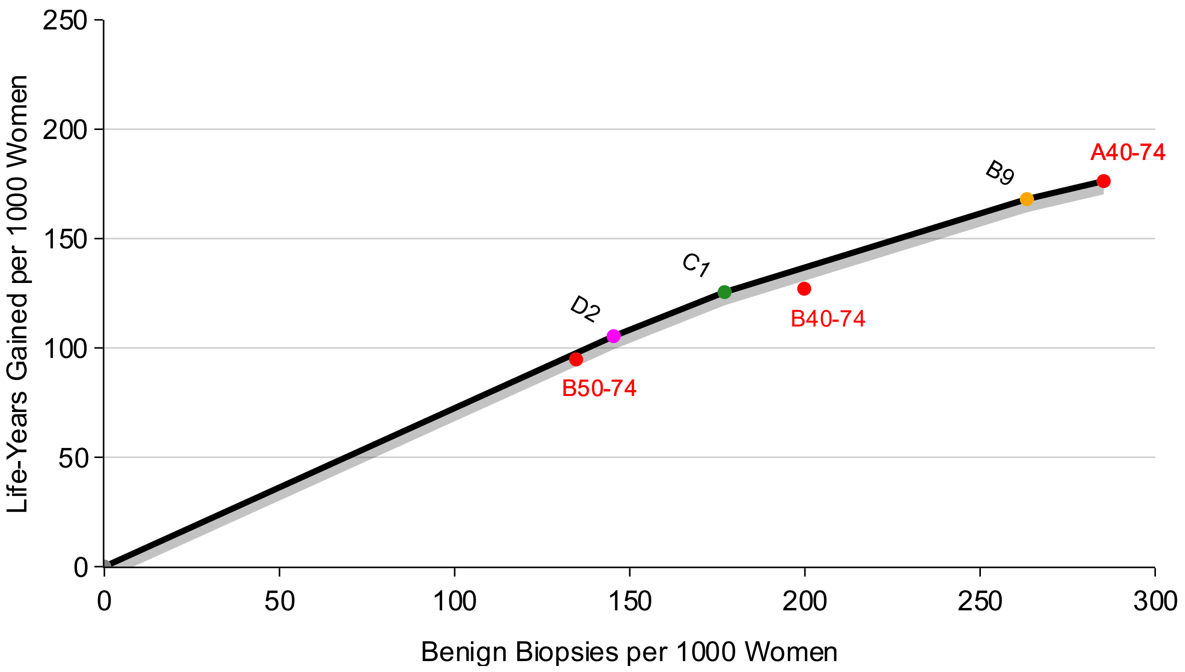

© 2026 Alagoz O et al. *JAMA Network Open.*

eFigure 8. Efficiency Frontiers Displaying all Policies for the Estimated Lifetime Number of False-Positive Mammograms, Life-Years Gained, and Breast Cancer Deaths Averted for a Cohort of 1000 Women by Screening Strategy. The outcomes in these graphs are obtained by taking the average of the outcomes estimated by model W and model E. A) Deaths averted vs. false-positives B) Life-years gained vs. false-positives

A)

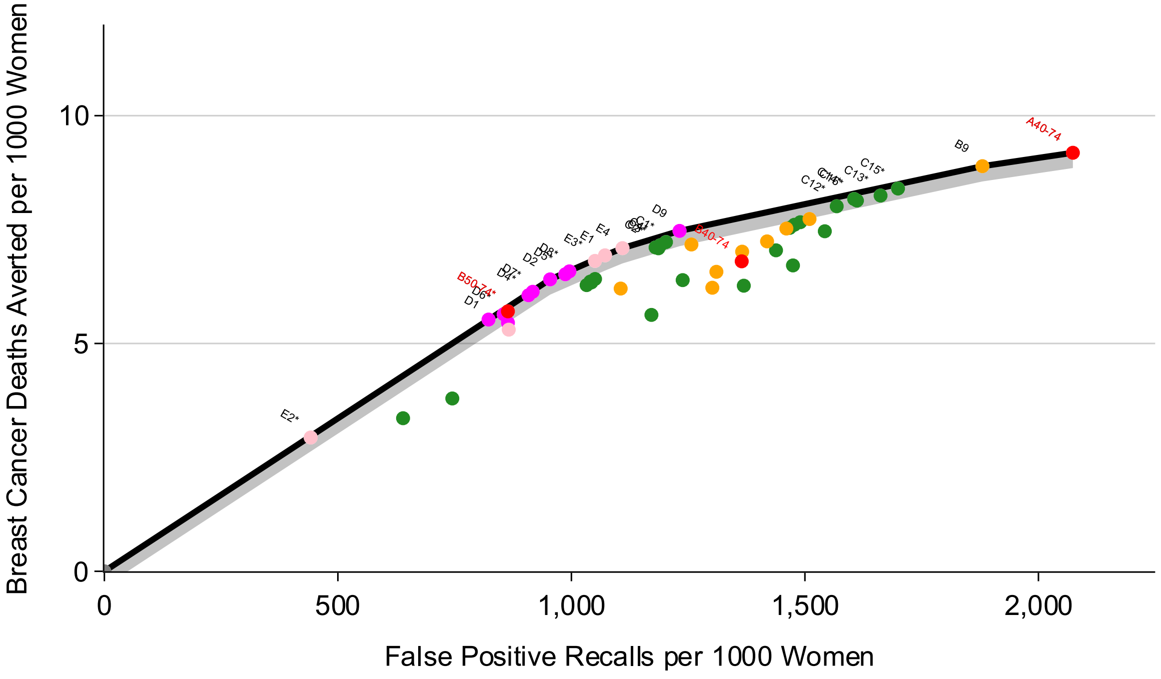

B)

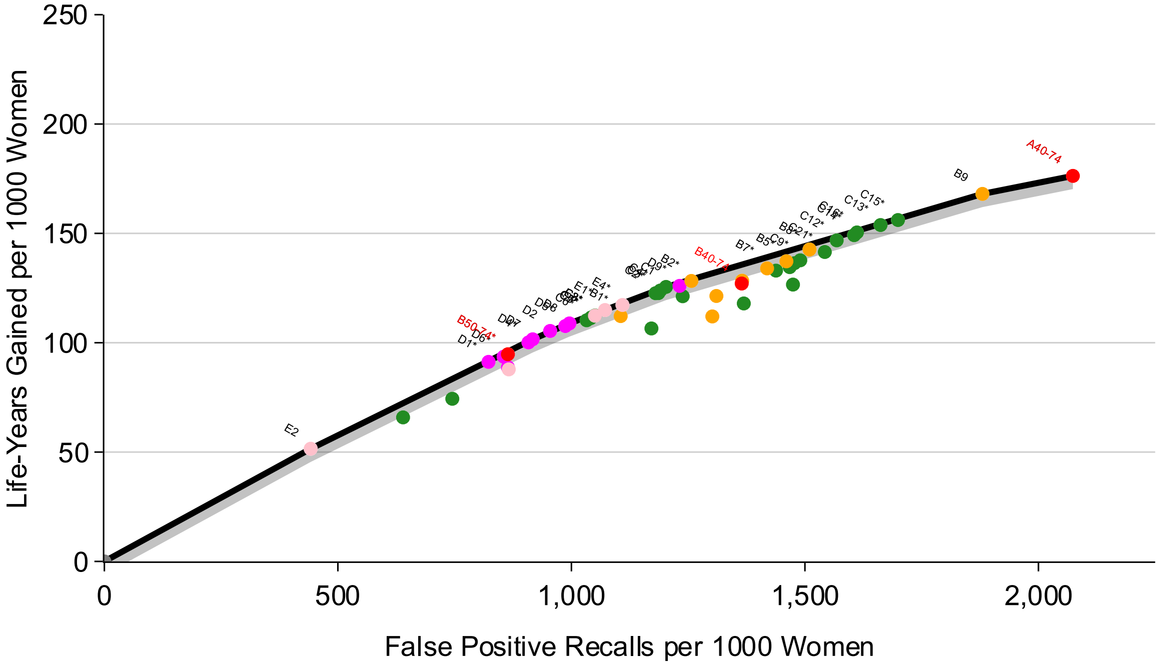

© 2026 Alagoz O et al. *JAMA Network Open.*

eFigure 9. Efficiency Frontiers Displaying all Policies for the Estimated Lifetime Number of Mammograms, Life-Years Gained, and Breast Cancer Deaths Averted for a Cohort of 1000 Women by Screening Strategy. The outcomes in these graphs are obtained by taking the average of the outcomes estimated by model W and model E. A) Deaths averted vs. false-positives B) Life-years gained vs. false-positives

A)

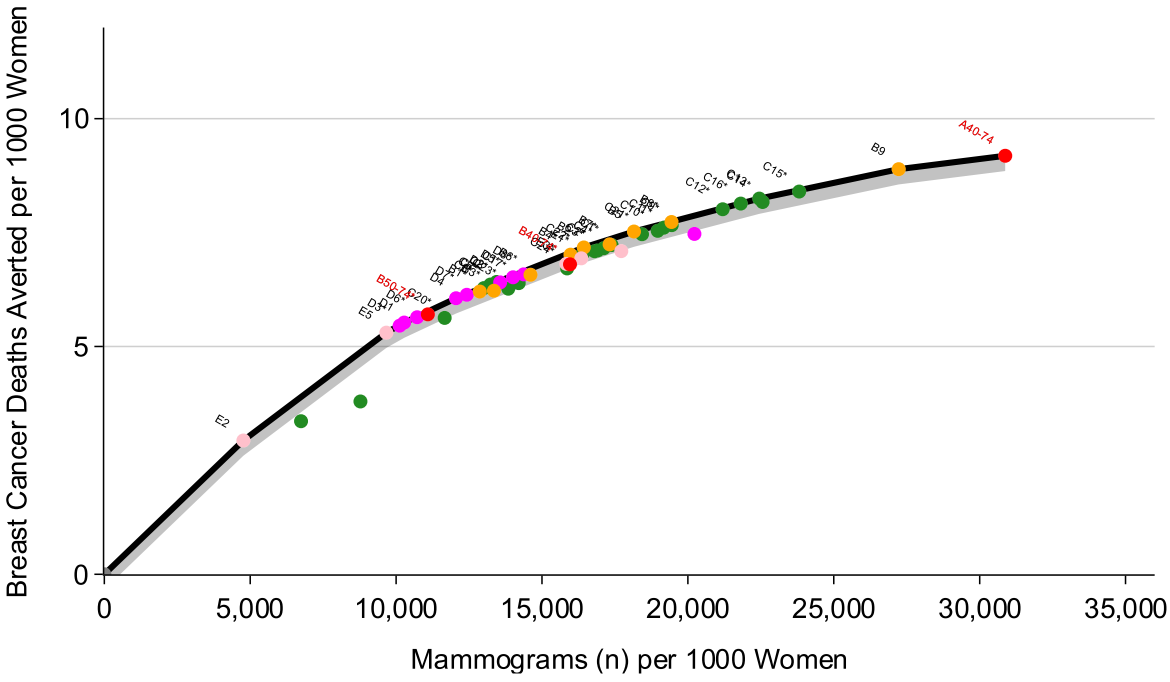

B)

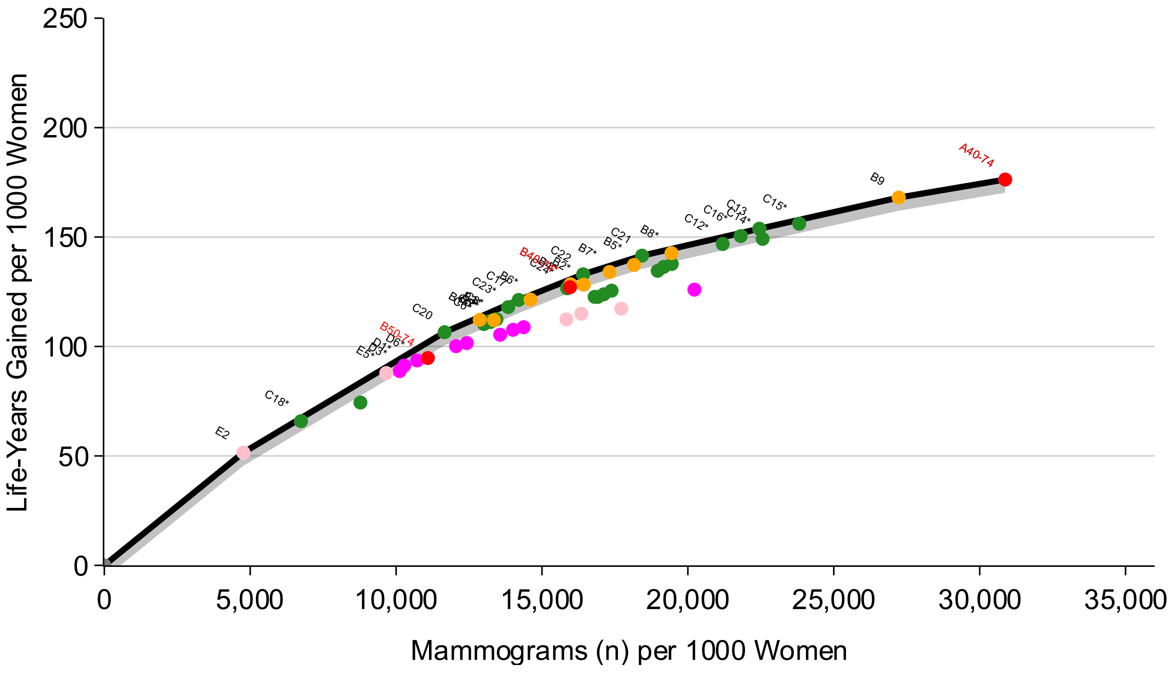

© 2026 Alagoz O et al. *JAMA Network Open.*

eAppendix 5. Impact of Initiation of Screening at Age 40 on the Projected Outcomes

In general, we found that earlier initiation of screening yields proportionally greater life-years gained (LYG) relative to breast cancer deaths averted. To further elaborate, we calculated the distribution of risk categories across age groups (40–49, 50–59, 60–69, 70–74) for strategies B2 and D9 (**eTable 9**). As the table shows, B2 recommends biennial and annual screening for 41% and 5% of women aged 40–49, respectively, thus conferring greater benefits to younger women. Consistent with this, earlier screening (B2 vs D9) produces proportionally more LYG (128 for B2 vs. 126 for D9) but fewer deaths averted (7.2 for B2 vs. 7.5 for D9), reflecting the greater relative benefit among women aged 40–49 who derive more life-years from early detection. On the other hand, although screening begins at a later age than B2, D9 results in a larger number of mammograms than B2 (20,224 vs. 16,434).

eTable 9. Distribution of Risk Categories Within Each Age Group and Distribution of Age Groups Within Each Risk Category for Strategies B2 and D9. Orange, yellow, and light-blue shaded cells represent no screening, annual screening, and biennial screening, respectively.

Strategy B2

Distribution of risk categories within each age group

Age group	Low-risk	Average-risk	Intermediate-risk	High-risk
40-49	55%	41%	4%	1%
50-59	18%	65%	11%	5%
60-69	2%	49%	24%	20%
70-74	1%	14%	35%	28%

Distribution of age groups within each risk category

Age group	Low-risk	Average-risk	Intermediate-risk	High-risk
40-49	73%	24%	8%	2%
50-59	23%	38%	21%	13%
60-69	3%	29%	42%	53%
70-74	1%	9%	28%	32%

Strategy D9

Distribution of risk categories within each age group

Age group	Low-risk	Average-risk	Intermediate-risk	High-risk
40-49	0%	0%	0%	0%
50-59	18%	66%	11%	5%
60-69	2%	53%	24%	20%
70-74	1%	35%	35%	28%

Distribution of age groups within each risk category

Age group	Low-risk	Average-risk	Intermediate-risk	High-risk
40-49	0%	0%	0%	0%
50-59	86%	51%	23%	13%
60-69	11%	38%	46%	54%
70-74	3%	11%	31%	33%

No screening	Biennial	Annual

© 2026 Alagoz O et al. *JAMA Network Open.*

eReferences.

1. Mandelblatt JS, Near AM, Miglioretti DL, et al. Common model inputs used in CISNET collaborative breast cancer modeling. Medical Decision Making. 2018;38(1_suppl):9S-23S.
2. Alagoz O, Lowry KP, Kurian AW, et al. Impact of the COVID-19 pandemic on breast cancer mortality in the US: estimates from collaborative simulation modeling. JNCI: Journal of the National Cancer Institute. 2021;113(11):1484-1494.
3. Holford TR, Cronin KA, Mariotto AB, et al. Changing patterns in breast cancer incidence trends. Journal of the National Cancer Institute Monographs. 2005(36):19-25.
4. Gangnon RE, Sprague BL, Stout NK, et al. The contribution of mammography screening to breast cancer incidence trends in the United States: An updated age-period-cohort model. Cancer Epidemiology, Biomarkers & Prevention. 2015;24(6):905-912.
5. Breast Cancer Surveillance Consortium. About the BCSC Available: https://www.bcsc-research.org/about. Accessed May 15, 2024.
6. Gangnon RE, Stout NK, Alagoz O, et al. Contribution of breast cancer to overall mortality for US women. Medical Decision Making. 2018;38(1_suppl):24S-31S.
7. Plevritis SK, Munoz D, Kurian AW, et al. Association of screening and treatment with breast cancer mortality by molecular subtype in US women, 2000-2012. JAMA. 2018;319(2):154-164.
8. Caswell-Jin JL, Sun LP, Munoz D, et al. Analysis of breast cancer mortality in the US—1975 to 2019. JAMA. 2024;331(3):233-241.
9. Kerlikowske K, Su Y-R, Sprague BL, et al. Association of screening with digital breast tomosynthesis vs digital mammography with risk of interval invasive and advanced breast cancer. JAMA. 2022;327(22):2220-2230.
10. Early Breast Cancer Trialists' Collaborative Group. Effect of radiotherapy after breast-conserving surgery on 10-year recurrence and 15-year breast cancer death: meta-analysis of individual patient data for 10 801 women in 17 randomised trials. The Lancet. 2011;378(9804):1707-1716.
11. Early Breast Cancer Trialists' Collaborative Group. Relevance of breast cancer hormone receptors and other factors to the efficacy of adjuvant tamoxifen: patient-level meta-analysis of randomised trials. The lancet. 2011;378(9793):771-784.
12. Early Breast Cancer Trialists' Collaborative Group. Comparisons between different polychemotherapy regimens for early breast cancer: meta-analyses of long-term outcome among 100 000 women in 123 randomised trials. The lancet. 2012;379(9814):432-444.
13. Early Breast Cancer Trialists' Collaborative Group. Effects of chemotherapy and hormonal therapy for early breast cancer on recurrence and 15-year survival: an overview of the randomised trials. The Lancet. 2005;365(9472):1687-1717.
14. Early Breast Cancer Trialists' Collaborative Group. Aromatase inhibitors versus tamoxifen in early breast cancer: patient-level meta-analysis of the randomised trials. The Lancet. 2015;386(10001):1341-1352.
15. Early Breast Cancer Trialists' Collaborative Group. Long-term outcomes for neoadjuvant versus adjuvant chemotherapy in early breast cancer: meta-analysis of individual patient data from ten randomised trials. The Lancet Oncology. 2018;19(1):27-39.
16. Mandelblatt JS, Stout NK, Schechter CB, et al. Collaborative modeling of the benefits and harms associated with different US breast cancer screening strategies. Annals of Internal Medicine. 2016;164(4):215-225.

© 2026 Alagoz O et al. *JAMA Network Open.*

17. Trentham-Dietz A, Kerlikowske K, Stout NK, et al. Tailoring breast cancer screening intervals by breast density and risk for women aged 50 years or older: collaborative modeling of screening outcomes. Annals of Internal Medicine. 2016;165(10):700-712.
18. Alagoz O, Ergun MA, Cevik M, et al. The University of Wisconsin breast cancer epidemiology simulation model: an update. Medical decision making. 2018;38(1_suppl):99S-111S.

© 2026 Alagoz O et al. *JAMA Network Open.*
