# Supplementary material for: Five-Year Absolute Risk–Based and Age-Based Breast Cancer Screening in the US
Source: JAMA Netw Open. 2026 Jan 20;9(1):e2552944. doi: 10.1001/jamanetworkopen.2025.52944 (PMC12820738; doi:10.1001/jamanetworkopen.2025.52944)
Supplement: Supplement 2. — Data Sharing Statement [file jamanetwopen-e2552944-s002.pdf]

Data Sharing Statement

Alagoz. Five-Year Absolute Risk–Based and Age-Based Breast Cancer Screening in the US. *JAMA Netw Open*. Published January 20, 2026. doi:10.1001/jamanetworkopen.2025.52944

Data
Data available: No

Additional Information
Explanation for why data not available: This is a modeling study, therefore, there is no patient-level data and all data used to generate the tables/figures are presented in the paper and the supplement.
